# RNN-Aided Human Velocity Estimation from a Single IMU †

**DOI:** 10.3390/s20133656

**Published:** 2020-06-29

**Authors:** Tobias Feigl, Sebastian Kram, Philipp Woller, Ramiz H. Siddiqui, Michael Philippsen, Christopher Mutschler

**Affiliations:** 1 Precise Positioning & Analytics Department, Fraunhofer Institute for Integrated Circuits (IIS), 90411 Nürnberg, Germany; sebastian.kram@iis.fraunhofer.de (S.K.); philipp.woller@iis.fraunhofer.de (P.W.); ramiz.siddiqui@iis.fraunhofer.de (R.H.S.); christopher.mutschler@iis.fraunhofer.de (C.S.); 2 Programming Systems Group, Friedrich-Alexander University (FAU), 91054 Erlangen-Nürnberg, Germany; michael.philippsen@fau.de; 3 Institute of Information Technology (Communication Electronics), Friedrich-Alexander University (FAU), 91054 Erlangen-Nürnberg, Germany; 4 Department of Statistics, Ludwig-Maximilians-University (LMU), 80539 Munich, Germany

**Keywords:** inertial navigation, motion tracking, velocity estimation, machine learning

## Abstract

Pedestrian Dead Reckoning (PDR) uses inertial measurement units (IMUs) and combines velocity and orientation estimates to determine a position. The estimation of the velocity is still challenging, as the integration of noisy acceleration and angular speed signals over a long period of time causes large drifts. Classic approaches to estimate the velocity optimize for specific applications, sensor positions, and types of movement and require extensive parameter tuning. Our novel hybrid filter combines a convolutional neural network (CNN) and a bidirectional recurrent neural network (BLSTM) (that extract spatial features from the sensor signals and track their temporal relationships) with a linear Kalman filter (LKF) that improves the velocity estimates. Our experiments show the robustness against different movement states and changes in orientation, even in highly dynamic situations. We compare the new architecture with conventional, machine, and deep learning methods and show that from a single non-calibrated IMU, our novel architecture outperforms the state-of-the-art in terms of velocity (≤0.16 m/s) and traveled distance (≤3 m/km). It also generalizes well to different and varying movement speeds and provides accurate and precise velocity estimates.

## 1. Introduction

Pedestrian dead reckoning (PDR) continuously estimates velocities and orientations to derive a stable position [1,2,3,4,5,6]. However, this only works well if both the accelerometer and the gyroscope are mounted on the feet [7,8] or on the spine [3,9,10]. Such methods then do not require additional external sensors or machinery that provide accurate positions over a longer period of time [11,12]. The technical challenges of sensor drift and the decomposition of the acceleration are still unsolved and even rotation-invariant methods that use frequency-based parameters still suffer from poor accuracy [7,10]. Instead, a highly precise initial calibration of the alignment of the sensors with respect to the environment is sufficient [5,6].

However, attaching the sensors to the feet or spine is not only unsuitable for everyday use, it is often impossible in many real-world use cases. Non-rigid mounting of the sensors (like e.g., in a user’s pocket) is very challenging for these approaches, as the orientation of the device w.r.t the tracked subject changes continuously and causes violations of the model [2,13,14]. Furthermore, these models are restricted to, and optimized for, specific motion types with little variation in velocity; they cannot be applied to jogging, running, and, especially, the transition between them [2]. To improve their accuracy dozens of motion models have been examined: models of empirical relationships [15,16], biomechanical models [15,17,18], linear models [19], non-linear models [20,21,22], and models that are based on regression [19,23,24,25]. However, they also suffer from orientation variance and provide less accuracy than data-driven state-of-the-art techniques [11,12,26].

PDR systems consist of two components: a heading and a velocity estimator. In this article, we focus on optimizing velocity estimation for complex and dynamic movement patterns and a non-rigid attachment of the sensors to the subjects’ bodies. Instead of modeling the complex relation between the measured inertial data and the subject’s velocity explicitly, latest methods [9,11,12,27,28,29,30,31] use machine learning (ML) and deep learning (DL) to estimate the velocity. However, these algorithms are trained and evaluated while using data with minor velocity and orientation variations. However, for applications, such as sports, it is desirable to have a velocity tracking method that can continuously track the subject’s velocity, even if there is a highly dynamic progression in motion states. From this follows that we need both a method and a representative dataset that generalize over different motion states.

We formulate inertial measurement unit (IMU)-based velocity estimation as a regression problem and then apply a novel hybrid filter architecture that combines DL (for velocity estimation) with a Bayesian filter (to optimize the estimation). A convolutional neural network (CNN) extracts spatial features from the signal stream. A recurrent neural network (RNN), specifically a bidirectional long-short-term-memory (BLSTM), uses the temporal relationships of the features to estimate robust, rotation-invariant, and highly accurate velocities. We use the magnitude of the gravity-free acceleration and the angular rate signals of a single, uncalibrated, low-cost IMU as typically used in consumer devices, e.g., smartphones. The article is an extended version of the approach published in [32].

We compare the novel hybrid architecture with state-of-the-art PDR, ML, and DL approaches. It learns to map the raw signal to a corresponding velocity, performs well, even with different and dynamically varying movement speeds and types (walking, jogging, running, and a random natural combination thereof), and also works robustly under dynamically varying orientations, since it only uses the signal magnitude vector (SMV). We evaluate the methods on temporally independent snapshots of a novel dataset labeled with a millimeter-accurate optical positioning reference systems and containing a variety of motion states and test subjects.

Our experiments show that, while classic approaches cannot generalize to different and varying motion types, our approach provides accurate and precise velocity estimates, specifically in challenging sports applications. Both the extension of the models with a linear Kalman filter (LKF) and the consideration of the temporal dependencies in the data improve the overall results. We show that our rotation-invariant architecture directly estimates velocities from the magnitude of raw accelerations and angular rates and outperforms the state-of-the-art in terms of the accuracy of the instantaneous velocity (≤0.10 m/s) and the distance covered (≤29 m/km) [33,34], even in highly dynamic scenarios.

Section 2 reviews related work. Section 3 introduces both our processing pipeline and our novel architecture. Section 4 deals with data acquisition and introduces the setup of an extensive measurement campaign. Section 5 evaluates our methods and discusses the results.

## 2. Related Work

As IMU-based PDR has been extensively studied by various communities (see [35,36] for a thorough overview), we focus on the most recent approaches that make use of ML.

Data-driven methods of human movement have been studied for decades, with focus on analyzing or reconstructing the gait and posture of walking people using inertial measurements [37,38,39,40,41,42,43]. Chang et al. [44] learn intuitive physics, Karl et al. [45] create state-space models, and Stewart et al. [46] monitor neural networks (NN) via physical knowledge. Others [8,47,48] learn to limit a system drift or to get a more precise zero velocity update (ZUPT) phase to compensate for the errors of inertial systems [11,12,49]. However, most of these approaches focus on the analysis of human movement and do not provide velocity, orientation, or position. While our method is inspired by known data-driven methods of analyzing human motions, we focus on localization that is based on velocity, that is, we implicitly first *extract* features of human motion with a CNN and then *track* them over time with an RNN.

Multiple Sensors. In the field of human localization, most DL methods are based on visual-inertial odometry (VIO) and combine different sensors [50]. Konda et al. [51] propose a CNN and use data from two synchronized sensors to predict the direction and the velocity changes. Carrera et al. [52] use two or more IMUs or a military-grade IMU [12] to reduce accumulating errors and increase the confidence of their algorithms. Others learn intuitive physics [3], design state space models [53], or monitor NNs via physical knowledge [46,54]. Because they rely either on a tightly coupled sensor fusion [55] or on the availability of reliable context information, such as external sensors [7,34,55,56] or maps [33,52,57], their accuracy is highly dependent on external information sources. Instead, our method works with a single inertial sensor and, therefore, does not suffer from error propagation from potentially incorrect external information.

Hybrid Methods. Hybrid methods [1,8,30,31,58,59] combine ML or DL with certain models, such as ZUPT or they combine classification and regression models to estimate both step length and orientation. A combination with ZUPT [8,11,60] reduces the sensor drift (error < 0.25 m/s) [8,57] and, hence, provides a higher accuracy. However, at higher movement speeds, ZUPT fails due to noise and movement artifacts [8,11,12,58]. Other methods first classify a movement type with ML or DL methods and then select a suitable step length estimator. For example, RIDI [31] splits the velocity and orientation estimation into three parts: first, a support vector machine classifies the placement of the sensor, and then RIDI uses the classification to select an optimal support vector regressor (SVR) to estimate velocities. Finally RIDI relies on traditional sensor fusion methods to estimate orientations. RIDI shows promising results (7.64 m versus PDR: 10.28 m on average) on the publicly available datasets RIDI [31], OxIOD [59], and RoNIN [29]. However, the classification of the sensor placement requires hand-crafted features and time-consuming training steps for each of the individual models. Even small anomalies in the signals mislead such hybrid methods [1,12], which leads to a low accuracy [31]. Hence, when flexibility of use is important for a real application, in general such methods cannot adapt easily. In contrast to these hybrid methods, we train our DL-based method in a supervised end-to-end manner on a variety of human movements with a large range of velocities and gaits.

Purely DL-based Methods learn end-to-end motion representations directly from raw data. Xing et al. [25], Cho et al. [61], and Martinelli et al. [62] use a simple NN to estimate the step length from consumer-grade inertial data. Their performance reaches that of naive SINS. To improve, recent studies use RNNs to de-noise a signal [30,63,64], to detect steps [1,8,58], or to estimate their length [1,8,59]. However, by splitting up the problem, the networks have to deal with interdependent errors created by the other system components. Hence, Wang et al. [26] combine two DL architectures to estimate the step length from noisy inertial sensor data: a long-short-term-memory (LSTM) to extract the time dependencies and a de-noising auto-encoder (DAE) to remove the noise from significant features. On average, they achieve a step length error of 4.63% and a distance error of 14.3 cm/m when a user walks slowly and with a spine-attached IMU. The combination of the LSTM and the DAE not only requires a lot of computing, but it is also rotation-variant. To estimate accurate positions, the user must hold the sensor in front of his/her chest. In contrast to these pure DL-based methods, our method does neither require a specific placement nor a rigid mounting of the sensor on a specific body part.

To address all of these challenges, Chen et al. [30] and Yan et al. [29] propose two DL models (IONet and RoNIN) that offer state-of-the-art positioning performance. IONet directly reconstructs trajectories from six-axis inertial data. A BLSTM network regresses both velocity and orientation. Instead, RoNIN divides the reconstruction of a trajectory into an LSTM network that estimates the orientation and a residual network (ResNet-18 [65]) that estimates the distance. Yan et al. [29] compared the performance of RoNIN, IONet, and PDR on public datasets [29,31,59]. When users walk in circles or on quasi-random paths, IONet suffered from even higher position estimation errors (14.60 m) than a classic PDR method (10.28 m), while RoNIN not only achieved state-of-the-art errors (6.71 m), but also surpasses the other methods w.r.t. accuracy (3.04 m). While IONet’s BLSTM architecture is an optimal temporal feature tracker, it is not an optimal spatial feature extractor. Additionally, since RoNIN is based exclusively on a CNN, it only takes spatial features into account, but cannot capture temporal dependencies [29]. This limits their ability to generalize to unknown environments [30,66] and the influence of long-term drift in velocity and orientation on their estimation performance remains unclear.

In contrast to IONet and RoNIN, our own earlier work [32] suggests a sequential combination of CNNs and BLSTMs. This model is rotation-invariant, as it works directly on the SMV of raw inertial data. The CNN first extracts spatial features, and the BLSTMs then captures the temporal emergence of these features to embed the entire motion dynamics of the inertial measurements. This accurately estimates velocity, especially in unknown motion scenarios with abrupt changes in movement and orientation and with various motion activities, such as standing, walking, jogging, running, and a combination, on random trajectories. In contrast to our earlier work, we replace the one-dimensional (1D) CNN architecture with a 1D ResNet-18 [67] architecture (similar to RoNIN [29]), merge it with a BLSTM (similar to IONet [30]), and then optimize the output with a LKF.

## 3. Method

Our novel processing pipeline combines three steps: the data pre-processing, the main processing with the DL architecture, and the optimizing post-processing step with a LKF. The pre-processing synchronizes the input data streams (*acc*, *gyr*, and *ref*), cleans them up, prepares them (by resampling and interpolation), and then bundles the data streams so that later on different models (classic, ML, and DL) can use them to estimate velocities $v^{\prime }$ based on the corresponding reference velocity $v_{ref}$. In the post-processing, a LKF returns velocities $v^{\prime \prime }$ that are even further optimized.

### 3.1. Data Pre-Processing

**Figure 1:**
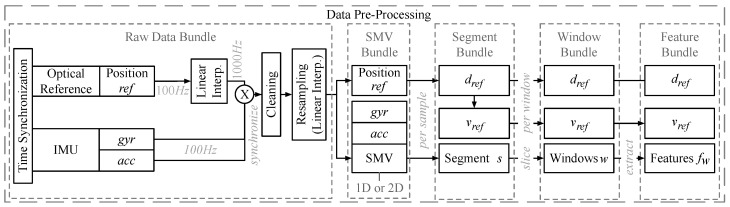
A zoomed-in view of the pre-processing pipeline.

Figure 1 sketches the data pre-processing in detail. The data flows from the input sensors (left) to the pre-processed data bundles (segments to features, on the right). The pre-processing is done per bundle, i.e., the data for a single activity of a single user.

Interpolation and Resampling. Pre-processing starts from the bundle of raw input data (from accelerometer acc, gyroscope gyr, optical reference system *ref*). We resample the reference positions *ref* to 1000 Hz. Subsequently, we synchronize them with the measurements of *acc* and *gyr*. To clean up the synchronized measurements, we visually and statistically detect corrupt data instances and outages and then remove them. We fix short dropouts (<20 ms) by re-sampling and re-interpolating. To generate several datasets for our benchmarks, we re-sample the clean measurement sets at 50, 100, 200, and 400 Hz. Section 4.2 discusses the configuration that offers the highest accuracy of velocity estimation. Afterwards, we calculate the SMVs of the data. We evaluate both the SMVs of the individual sensors (two-dimensional (2D): acc, gyr) and a combined SMV of both sensors (1D: acc + gyr).

**Figure 2:**
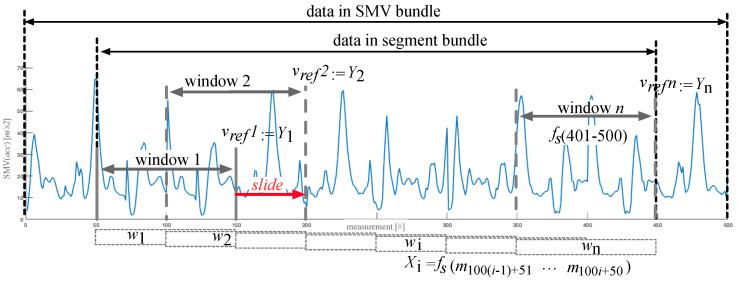
Exemplary cutout of a segment that shows an SMV(acc) of a single walking activity. We slice windows from a clean segment and calculate the reference velocities at the end of each window.

Bundling. From the SMV bundle, we create the segment bundle to evaluate the classic baseline PDR method. While the SMV bundle represents all of the initial raw data bundle, the segment bundle only holds the segment of the data that matters for the user’s activity, i.e., we remove the initial and the final parts (that may have been accidentally recorded before and after the activity) and only keep the repetitive stretches of the movement, see Figure 2 for an example. We calculate $d_{ref}$ and $v_{ref}$ by differentiating the 2D velocities, i.e., the translational layer spanned by the *x*- and *y*- axes obtained from the reference system (w.r.t. time and norm). From there, we create the window bundle to evaluate state-of-the-art DL methods and our hybrid DL method. The window bundle is a representation of the data in the corresponding segment bundle as a set of consecutive windows. We consider both overlapping and non-overlapping windows, see the paragraph on parameters below. Figure 2 sketches the slicing process of a segment into a bundle of windows. From there we create the feature bundle to evaluate the ML baseline method. For each of the windows in a window bundle the feature bundle holds the features $f_{w}$ that we extract from the data, see Section 4.3 for details.

Dataset Variants and Parameters. We use three variants of these dataset bundles. They will be discussed in detail in Section 4.2. We also evaluate different sampling rates $F_{s}$ (50, 100, 200, and 400 Hz) and different window sizes $N_{w}$ (64, 128, 256, and 512 Hz) of acc and gyr to find combinations of $F_{s}$ and $N_{w}$ that are computationally efficient and provide accurate velocity estimates. Additionally, when we slice a segment bundle into windows, there is the option to let windows overlap by $N_{w}/2$. We generate window bundles with length 32, 64, 128, and 256 Hz for each segment bundle. Finally, for all variants and parameters, we split all of the segment bundles, window bundles/groups, and feature bundles/groups into training, validation, and test datasets and then train our estimators on labeled data. At run-time, we predict velocities from unknown input data using the same pipeline. We do this independently for *V1*, *V2*, and *V3*. Further details on the datasets are covered in Section 4.2.

Before we introduce the details of our DL architecture, we introduce some important notations and definitions. Below, the *x*-axis, Figure 2 shows how we turn the windows from the pre-processing into sequences that serve as input into the DL network. In the example, window *w* holds 100 measurements m, taken at 100 time steps. While, so far, we have worked on the measurement data and have used the index *i* to refer to timestamps, we now work on the granularity of full windows, so that the index *i* refers to the number of a window. $X_{i}$ then represents the window $w_{i}$ with all of its m measurements. This is the input of the NN. It holds all the spatial features $f_{s}$ for m∈[100·(i−1)+51,100·i+50]. $Y_{i}$ is the output of the NN, i.e., $v_{ref}$ during the training phase of the network or the predicted velocity $v^{\prime }$.

### 3.2. Main Processing

Background. NNs, such as multi-layer perceptrons (MLPs), consist of an input layer *X*, one or more hidden layers h, and an output layer *Y*. Input signals propagate through the network, a loss function compares the output to the ground truth label to calculate the error, error signals propagate backwards through the network, and weights are adjusted in order to reduce the error [68]. All of the MLPs only take a fixed set of input measurements m, e.g., acc and gyr samples, per time step *i* (a snapshot). Hence, they cannot describe the relationship of time and context (temporal features $f_{t}$) over successive inputs to predict a velocity $v_{i}$. Instead, they describe spatial features $f_{s}\left(X_{i}\right)$ of a snapshot that have no connection or relationship to a previous $f_{s}\left(X_{i-1}\right)$ or successive snapshot $f_{s}\left(X_{i+1}\right)$. However, we state that these relationships ($f_{t}$) are relevant, as they represent changes over time, especially in highly dynamic motions sequences.

RNN. We use time- and context-sensitive NNs such as LSTM or BLSTM to learn dependencies of $f_{s}$ in a single window and their short-long-term dependencies $f_{t}$. They capture (1) the relationship of each measurement $m_{j}$ in a window $w_{i}$ to all other $m_{k}\in w_{i}$ in the same window (i.e., how each measurement spatially affects other measurements in the same window) and (2) memorize their dependencies and effects, i.e., how each measurement affects the measurements over a long period of time, e.g., how a motion $f_{s}$ with the same velocity for all users has varying $f_{t}$ between them [64]. To be comparable to the state-of-the-art and the baseline methods, we process input sequences with a single window. To reconstruct latent connections between motion characteristics and data characteristics, a BLSTM takes advantage of temporal dependencies $f_{t}$, as it maintains its hidden states over the duration of a window. It also exploits the dynamic context and examines the current window from both the past and the future. It effectively acts as a function that maps sensor measurements to a corresponding velocity. In our results, see Section 5, we show that this type of RNN in combination with a state-of-the-art feature extraction can take advantage of long-term dependencies $f_{t}$ in batches and that the lack of $f_{t}$ leads to significantly worse results.

**Figure 3:**
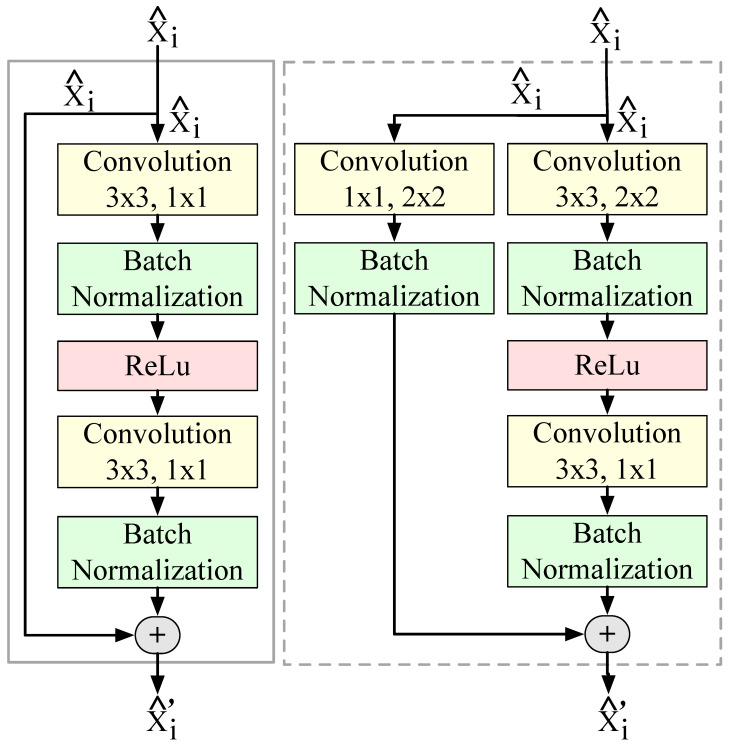
Architecture of residual units.

Feature Extraction. While RNNs track signal characteristics well over time, we must derive such characteristics from raw signals. An obvious approach is to use hand-crafted features (which we use for the ML-methods, see Section 4) and then use them as input for the RNN. CNNs, proved to be much better spatial feature extractors (although they cannot put these features in a temporal context). Feigl et al. [32] propose a shallow 1D CNN architecture to extract $f_{s}$ from 1D inputs. It is well known that wider or deeper CNNs can extract more characteristic and representative $f_{s}$ that result in a higher accuracy of the estimates [69]. However, the deeper the CNN is, the more it suffers from a vanishing gradient and the higher is its computing load [70]. Residual networks with multiple convolution layers (such as ResNet-18 [65]) solve this problem. Their extremely deep networks do not suffer from vanishing gradients and they are both accurate and computationally efficient in various applications. Regardless of the network type, the gradient ${\hat{X}}_{i}$ is duplicated first, as shown in Figure 3. While type I networks convolute the features ${\hat{f}}_{s}$ in only one of the copies, type II networks convolute both of them before in the end they add up both results. Thus, type II residual units re-parameterize the convolutions (e.g., stride length s, filter kernel size $F_{s}$, and the number of filter kernels *F*). Both types also have batch normalization (BN) layers (between convolutional layers) and rectified linear unit layers (ReLUs) to speed up training and reduce the sensitivity to initialization. In general, residual networks start with a moderate number of features $f_{s}$, at the input ( = $X_{i}$). Further down, the dimensionality of $f_{s}$ grows (for certain configurations up to about 10 M). Note that in the above description we used ${\hat{X}}_{i}$, ${\hat{X}}_{i}^{\prime }$, and ${\hat{f}}_{s}$ for inputs, outputs, and features that propagate through the NN. We use the ^ to indicate intermediate values. Sizes, dimensionality, and feature sets usually differ significantly between layers.

**Figure 4:**
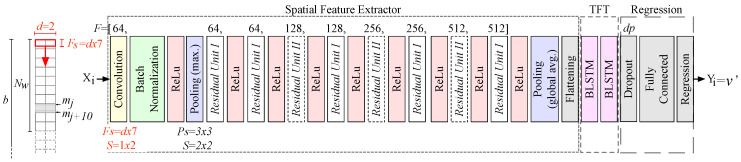
Detailed overview of our deep learning (DL) architecture: The deeper the network, the more spatial features (from 64 to 512) we extract. The two BLSTM layers track the temporal emergence of $f_{s}$ in the “temporal feature tracker” (TFT). The Residual Units of type I and II are also depicted in Figure 3.

Architecture. We use a modified ResNet to extract high-dimensional ${\hat{f}}_{s}$ from the raw input signals (a single window of $f_{s}\left(X_{i}\right)$ for each time step *i*), which is then fed into a so called “temporal feature tracker” built from two BLSTMs. Figure 4 shows the architecture. Note that the input flows from left to right, i.e., from input $X_{i}$ ( = $w_{i}$) to output $Y_{i}$ ( = $v_{ref}$). In contrast to the original ResNet-18 [65], (1) we adapt the initial convolution layer to our input dimensions; (2) at the end of the so called “spatial feature extractor” we replace the Softmax and classification layers with a flattening layer; and (3) we add two extra BLSTM layers, a fully connected (dense) layer, and a final regression layer.

The initial convolution layer applies 64 striding convolution filters (*F* = 64) with the size $F_{s}$ = d×7 to the input $X_{i}$ from $w_{i}$. It strides each *F* vertically (with stride length S=1×2) through the input. Figure 4 shows this in red for d=2, S=1×2, and $F_{s}=2\times 7$: it uses 14 measurements from a window and then calculates the dot product of the weights and the measurements and then adds a bias term. The convolution layer emits a multi-dimensional set of values ${\hat{X}}_{i}$ and features ${\hat{f}}_{s}$. A BN layer then normalizes each input channel across the complete batch of inputs. As activation functions, we apply a ReLu layer that zeros out any elements of the input that are below zero to add non-linearity to the network. Our first pooling layer divides the input into rectangular pooling areas (with size P=3×3) and then calculates the maximum of each region to sample the input down (i.e., to reduce the spatial size of the representation and, thus, to reduce the number of parameters and calculations). Subsequently, we concatenate several residual layers of types I and II to increase the number of potential internal features ${\hat{f}}_{s}$ (from 64 to 512). A global max pooling layer calculates the maximum of the height and width dimensions of the input to sample it down, i.e., to reduce ${\hat{f}}_{s}$ to only keep prominent features. A flattening layer provides a flat sequence of ${\hat{f}}_{s}$ to fit the input dimensions of the first BLSTM layer. It collapses the high spatial dimensions of the input ${\hat{f}}_{s}$ into the channel dimension ${\hat{x}}_{i}$ of our BLSTM (i.e., from the multi-dimensional output of the pooling layer ${\hat{f}}_{s}$ to the 1D input ${\hat{x}}_{\left(1,n\right)}$ of the BLSTM layer).

**Figure 5:**
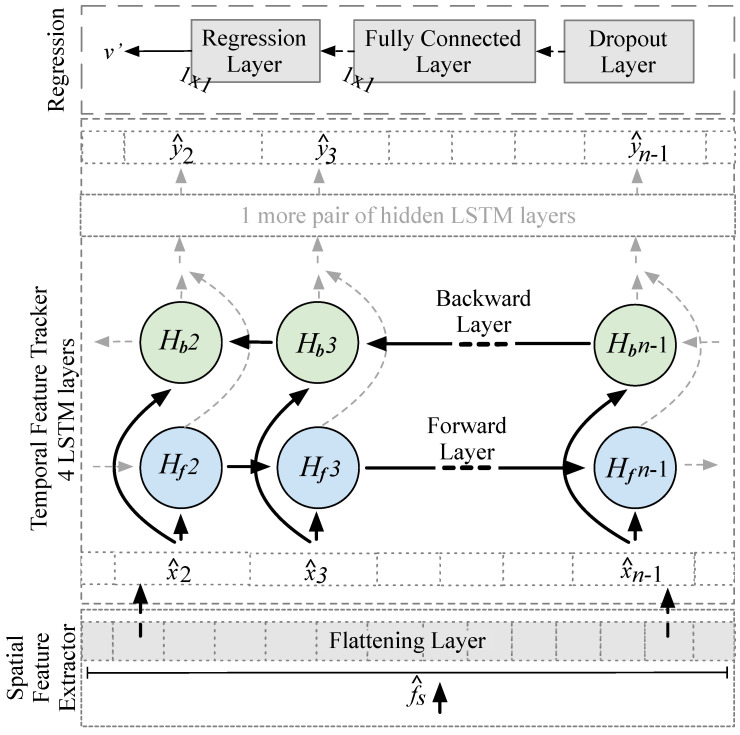
“Temporal feature tracker” built from two BLSTMs, each with two hidden *f*orward ($H_{f}$) and *b*ackward ($H_{b}$) long-short-term-memory (LSTM) layers (only one shown; no LSTMs for ${\hat{x}}_{1}$ and ${\hat{x}}_{n}$).

Figure 5 sketches the tail of the architecture, from the flattening layer to the BLSTM network. $\hat{x}$ and $\hat{y}$ (lowercase letters) are the input and output of the BLSTM layers. For the first BLSTM layer $\hat{x}$ is the flattened representation of the ${\hat{f}}_{s}$, i.e., there is another change of the representation (${\hat{x}}_{1}$ to ${\hat{x}}_{n}$ represent the flattened ${\hat{f}}_{s1}$ to ${\hat{f}}_{sn}$). The two BLSTM layers learn bidirectional long-term dependencies $f_{t}$ between the ${\hat{f}}_{s}$ that we extract with ResNet (i.e., between different motions that represent the same velocity). Each of the two BLSTM layers consist of a single hidden *f*orward LSTM layer ($H_{f}$) and *b*ackward LSTM layer ($H_{b}$). Figure 5 only shows the first two of the four LSTM layers. To indicate the capability of BLSTM to look at the data from both the past and future, there are no LSTM cells for ${\hat{x}}_{1}$ (“the past”) and ${\hat{x}}_{n}$ (“the future“) but we left the gray arrows in place. The last BLSTM layer acts as a bottleneck and yields the last ${\hat{y}}_{n}$, which is processed through a dropout layer to prevent overfitting (dp = 0.5 = 50% defines the number of dropped elements). A fully connected layer connects each of its neurons to all activations in the previous layer and a regression layer returns the velocity $v^{\prime }$ = $Y_{i}$ that we optimize while using a half-mean-squared-error loss.

Design Decisions. We design our ResNet18-BLSTM architecture in such a way that it first extracts and generates high-dimensional features $f_{s}$ from convolution layers and then tracks their temporal coherence (i.e., the context $f_{t}$). It memorizes the evolution of feature maps that represent the mapping of motion to velocity) in the BLSTM layers. The filter kernels *F* of our 1D ResNet-18 extract $f_{s}$ as they stride over a batch of windows to detect features at different positions [71]. The idea behind the 1D convolution is to perform an element-by-element multiplication for each 1D filter kernel with every 1D window to create an initial feature map that is sensitive to time [72]. As soon as the first part of the network has extracted high-level features ${\hat{f}}_{s}$ from the window bundle, they are put into BLSTMs. The added pooling reduces the complexity of the network and focuses on the most promising ${\hat{f}}_{s}$. Unlike simple LSTMs, our BLSTM processes information regarding the future and the past by retrieving the short-term and long-term context between input and output in both the forward and backward directions [71]. The BLSTM acts as a time- and context-sensitive detector and tracker of high-level motion features (extracted by the convolution layers) in the signal embedding (acc and gyr). We use the last hidden layer as the bottleneck layer [72]. From a single window m, the model learns how to estimate a single velocity $v_{i}^{\prime }$ and how to predict consecutive ones. Because the initial state $H_{0}$ is randomly initialized, the model has no previous knowledge of the movement, and thus learns to estimate the velocity from local information. We select the last velocity estimate ${\hat{y}}_{n}$ as the final result $v_{n}^{\prime }$ per window.

### 3.3. Post-Processing

The post-processing uses a LKF to optimize the velocity $v^{\prime }$ that our RNN predicts. The LKF [73,74] is known to reduce noise artifacts in temporal information, such as velocity. It consists of linear state transition models and measurement models and it assumes that all noise components are a non-zero additive Gaussian noise *c*. The state transition model describes the dynamics of a moving object, e.g., the IMU sensor. The measurement model describes the relationship between the tracked state (optimized velocity $v^{\prime \prime }$) and the expected measurements (input velocity $v^{\prime }$). The covariance of the state transition noise $c_{v,d}$ assumes the reliability of the state. Based on this reliability, the predictions and measurements hold information regarding the filter weights. The filter tracks the object’s velocity $v^{\prime \prime }$ as a state. Our state transition model assumes a constant velocity between successive time steps *i* and i+1:

(1) $$\begin{array}{c} v_{i+1}^{\prime \prime }=v_{i}^{\prime \prime }+c_{v,d}, \end{array}$$

with the covariance of the state transition noise $c_{v,d}∼N\left(0,\;T^{2}\sigma_{v,d}^{2}\right)$ and the time interval of the sampling *T* (= t(i+1)−t(i)). Because the filter only processes the velocity $v^{\prime \prime }$ as a 1D state, we can define the state transition with a scalar and set the state transition covariance to $T^{2}\sigma_{v,d}^{2}$. We estimated $\sigma_{v,d}$ by calculating the standard deviation of the derivative w.r.t. time of the velocities on the validation data. Typically, the model’s assumption of validity decreases over time. Similar to the state transition model, our measurement model also assumes additive white Gaussian noise $c_{v,m}∼N\left(0,\;\sigma_{v,m}^{2}\right)$:

(2) $$v_{i}^{\prime \prime }=v_{i}^{\prime }+c_{v,m}.$$

Again we define our measurement model with a scalar, but we set the measurement covariance to $\sigma_{v,m}^{2}$. We compute the error standard deviations of $v^{\prime }$ on the validation dataset to determine $\sigma_{v,m}$ for each (main processing) method individually, and then compute the standard deviation of the velocity derivative (corresponding to the prediction model error) of the validation data to obtain $\sigma_{v,d}$. We use these parameters (given directly from the data) in a standard LKF implementation [73] to predict $v^{\prime \prime }$ on $v^{\prime }$ at every time step *i*.

## 4. Experimental Setup

We describe the components of the experimental setup (Section 4.1) and the data acquisition process (Section 4.2). We next discuss the variants of the dataset (Section 4.2). Finally, we provide details regarding the parameterization of the classic, the ML, and the DL methods (Section 4.3).

### 4.1. Hard- and Software Components

**Figure 6:**
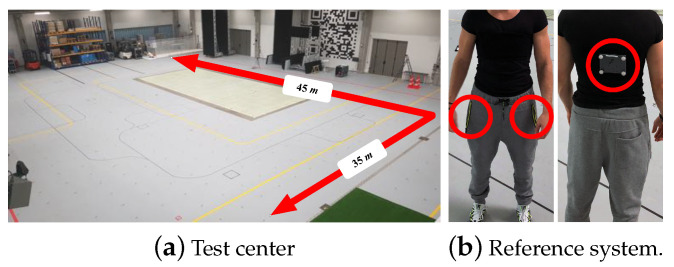
Setup.(**a**): lateral view of the data acquisition environment; (**b**): smartphones in the pockets of a trial subject and trackable object of the reference system.

Measurement Area. We recorded the motion data (i.e., acc and gyr) at the Fraunhofer IIS L.I.N.K. (localization, identification, navigation, and communication) test center in Nürnberg, Germany. It provides a unique test ground on 1400 m${}^{2}$, see Figure 6a [75], equipped with a variety of positioning reference systems. We used the following measurement and reference systems to collect and label our training, validation, and test data, and to capture the characteristics of different high dynamic motion types.

Reference System. We recorded refe-rence data (3D positions) with 28 cameras of the millimeter-accurate optical Qualisys motion tracking system (spherical error probable ($SEP_{95}$) ≤5 mm and ≤0.1°). The cameras are mounted on the edges of the upper side of the test area and cover a volume of 11.025 m${}^{3}$ (45 ×35× 7 m). The subjects wore four small trackable reflective markers, attached to an elastic ribbon, see Figure 6b. All of the cameras had a clear field of view when we tracked each subject’s position and orientation with constant 100 Hz.

IMU Measurement Device. We used two Samsung Galaxy S7 phones with their acc and gyr sensors (STMicroelectronics LSM6DS3 samples acc at ±16 G and gyr at ±1000 dps at quasi constant 100 Hz) to measure the subject’s accelerations and angular rates. To cover motion differences between the legs, we loosely placed a phone (inverse portrait) in each of the pockets, see Figure 6b. Because we evaluate all of the methods on the SMV of the signal, the input data are rotation-invariant. Hence, we do not care about the initial location and orientation. Note that we used two phones to collect more data per subject in parallel. However, we train and predict velocities on data from only one sensor.

IMU Software and Time Synchronization. We accessed the raw IMU sensor data of the smartphones via the Android API (Version 6, 2019) [31]. We stored them along with timestamps that are globally NTP-time-synchronized. Our API also received the NTP-time-synchronized 3DoF and 6DoF state information data as streams from Qualisys via 5GHz WiFi. We labeled each activity and measurement (acc, gyr, and ref) and stored separate files per subject. Thus, we can also use our data for activity classification. Note that we sampled the reference data (from 100) up to 1.000 Hz to synchronize with them the inertial measurements (constant 100 Hz).

### 4.2. Data Acquisition and Datasets

**Figure 7:**
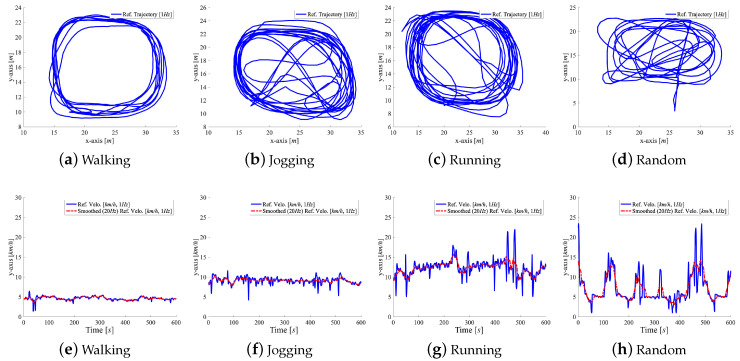
Exemplary segment bundle of one user. Each datapoint represents a single window. The upper row shows reference trajectories (at 1 Hz) and the bottom row shows $v_{ref}$ (at 1 Hz).

We asked 23 people (male: 17, female: 6, average age: 26.7, heights from 146 cm to 187 cm, SD 19 cm) to perform four different types of movement activities (walking, jogging, running, and random movements, a natural combination of all) within our tracking area. The participants stand still for 1 min at the beginning of each activity to enable estimation of initial sensor bias. Each subject and activity starts at roughly the same initial position. This helps to calibrate and identify the initial pose in repeated measurements. We manually labeled the activities (class according to activity). Each activity took 7.5 min on average (SD 1 min). The users performed the walking, jogging, and running activities at different velocities, but mostly along similar movement trajectories, see Figure 7. However, each participant performed the random movements in a unique way, i.e., changed between walking, jogging, and running at individual movement speeds and trajectories. The different types of running pants (with wide or tight pockets) introduced various sensor noise and motion artifacts.

**Figure 8:**
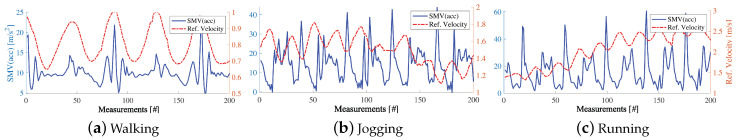
Raw acc signals: SMV (blue), $v_{ref}$ (red).

In total, we recorded data regarding 23 h = 1381 min of motion data (=4 activities × two sensors × 23 subjects × 7 min (SD 1 min)) with circa 30 min per sensor (two or one) per subject (SD ± 4.6 min). The full study resulted in a total traveled distance of around 107 km (about 214 km for two sensors per user) for all activities (walking: ~14 km; jogging: ~29 km; running: ~38 km; and, random: ~26 km) at different speeds (average for walking: 1.4$\frac{m}{s}$; min. 0.8$\frac{m}{s}$; max. 2.2$\frac{m}{s}$; SD 0.31$\frac{m}{s}$; jogging: 2.8$\frac{m}{s}$; min. 1.5$\frac{m}{s}$; max. 3.4$\frac{m}{s}$; SD 0.34$\frac{m}{s}$; running: 3.6$\frac{m}{s}$; min. 2.9$\frac{m}{s}$; max. 7.9$\frac{m}{s}$; SD 0.67$\frac{m}{s}$; random: 2.5$\frac{m}{s}$; min. 1.2$\frac{m}{s}$; max. 7.8$\frac{m}{s}$; and, SD 3.19$\frac{m}{s}$). Figure 7 shows an exemplary subset of 10 min of motion for a single user. The graphs show trajectories (top row) and velocities (bottom) of all activities (left to right). Each data point represents a single window without overlap. Figure 8 shows SMV acceleration (blue) and velocity (red) for walking, jogging, and running for 200 measurements. The signal complexity increases with higher velocities, peaks get less prominent. The complete data pool consists of 92 segment bundles (23 subjects × four activities) with 64,688 consecutive windows (without overlap, $N_{w}$ = 128, $F_{s}$ = 100 Hz approximately 350 windows per user, per activity, and per sensor) or 129,375 overlapping windows (50% overlap, $N_{w}/2$ = 128/2 = 64) with corresponding $v_{ref}$.

From this data pool, we created three dataset variants with sliding windows of size 128 Hz and an overlap of 50% ($N_{w}/2$ = 64), as there were enough prominent features ${\hat{f}}_{s}$ and ${\hat{f}}_{t}$ at low computational costs to achieve the highest velocity accuracy. 1.28 s cover long-term relationships of human movement and make our hybrid DL method applicable. Note that these parameters give the best results for variants V1, V2, and V3 of our dataset. The description of the dataset, the parameterization of the methods, and the results in Section 5 refer to this setting of the parameters.

Dataset Variant V1 provides segment, window, and feature bundles (276 = 3 × 92) that cover the entire data for every subject and every activity. Bundle sizes vary between users and activities, as, for example, some users jog at velocities that are the running speeds of others. With data from both sensors, V1 holds 129,375 overlapping (50% = $N_{w}/2$ = 64) windows or features at $N_{w}$ = 128 and $F_{s}$ = 100 Hz (approximately 700 windows per user, per activity, and per sensor with overlap).

Dataset Variant V2 provides segment, window, and feature bundles (276 = 3 × 92) trimmed to the shortest bundle size of all users. Hence, it consists of the same amount of individual movements per user and activity, i.e., the activities are distributed equally. However, the velocities are again not distributed equally. In total, V2 contains 112,643 overlapping windows or features (50% = $N_{w}/2$ = 64) at $N_{w}$ = 128 and $F_{s}$ = 100 Hz (approx. 600 windows per user, per activity, and per sensor with overlap).

Dataset Variant V3 merges all activities of all users. It only offers window and feature bundles (184 = 2 × 92). We sort all of the windows of all users and all their activities from V1 w.r.t. the reference velocity $v_{ref}$. We then bin the windows with a bin width of 2 km/h, resulting in ranges [3,5), …, (11,13) km/h. For each of these ranges, we found a common total of 156 entries (per sensor) for all users and activities. We delete ranges with fewer than 156 entries and limit bins with more than 156 entries to 156 entries (we randomly delete surplus entries). This is in line with typical pre-processing techniques in statistical analysis and DL to analyze evenly distributed classes/categories in data and their impact on processing methods [76,77]. Therefore, V3 contains homogeneous velocity groups across all users, regardless of their activity. Note that, within a range, gait varies between participants, i.e., it is different how a movement leads to the same velocity and to the resulting features ${\hat{f}}_{t}$ that our data-driven model learns. V3 contains a total of 35,880 windows or features without overlap (five velocity ranges × 156 motion/velocity pairs per user × 23 users × two sensors without overlap).

### 4.3. Parameterization of Velocity Estimators

Baseline I, classic PDR. We use a state-of-the-art PDR method that implements a well-established biomechanical model of Tian et al. [16] and we generalize it for unknown users and movements. With inertial measurements, this PDR recognizes peaks as steps in the segment bundles and estimates the step length (with *L* = $K\cdot h\cdot \sqrt{fq}$, where h is the height of the person, *K* is a calibration parameter, and fq is the step frequency) from there. Existing PDR implementations, in general, differ in how they determine *K* and fq [20,78]. We saw the best results with the acc’s SMV, since it enables results in quasi-rotational-invariance. An important insight was (a) to use a Butterworth low-pass filter with a cut-off frequency of 15 Hz to remove high-frequency noise before spotting the peaks and (b) to ignore peaks below a threshold of 17.23 m/s${}^{2}$ for walking, 24.17 m/s${}^{2}$ for jogging, and 32.61 m/s${}^{2}$ for running. Since the exact relationship between step length and step times is specific for a particular person s and a certain activity *a*, such as walking and running, we use, inspired by Weinberg et al. [20], subject- and activity-specific (*K* = )$K_{sa}$ that we accurately calibrated $K_{sa}$ while using ground truth data that also provided the reference height h for each participant. We took the first steps of a person (approximately 1 min) performing each activity and determined the ratio of the estimated distance $d^{\prime }$ = $v^{\prime }\cdot dt$ (dt is the time span of two steps) and the reference distance $d_{ref}$ between two steps: $K_{sa}$ = $d^{\prime }$/$d_{ref}$. However, in realistic application settings, it is extremely tedious to calibrate $K_{sa}$ values per subject and per activity. Therefore, for a fair comparison to the other four estimators, we generalize the step length estimation by deriving a single general calibration parameter $K_{g}$, which embeds the movement characteristics of multiple users and activities. We averaged all 80 $K_{sa}$ parameters (20 users × four activities) $K_{g}$ = $\frac{1}{n}\sum_{1}^{n}K_{sa(i)}$ = 1.2771. Note that, using the specific coefficients $K_{sa}$, the accuracy of the PDR velocity estimation increased by 19%, but this did not at all change the results of the comparison in Section 5.

Baseline II, ML with Gaussian Processes ML-GP. To find the best ML method for the supervised velocity regression problem, we conducted a serious study that included a set of well-known ML-based methods, as well as a large set of handcrafted statistical and frequency domain features that are known to work best on similar tasks [79]. The study optimized in a grid search the parameters of the methods and evaluated various combinations of features to find the one method and feature set that yield the highest accuracy of ML-GP. See Appendix A.1 for details on the features that we investigated [80]. On the datasets V1, V2, and V3, this resulted in 84 feature combinations for each window (42 feature bundles = six statistical × seven frequency domain features; plus the two largest principal components (PCs) of each of these feature bundles. The search also considered various regression methods (proposed by Bishop et al. [79]) for all possible combinations of the features mentioned above: linear regression, logistic regression, classification and regression tree (CART), SVR, and Gaussian process regression (GP) [81,82]. By means of a grid search for these five regression methods and the 84 combinations of features we derived the optimal hyper parameters, see Appendix A.2 for details. Among the estimators, GP delivers the highest accuracy with a Matern52 kernel. Interestingly, Matern52 achieves the highest accuracy with only the two PCs that represent the strongest characteristics of the complete set of all 42 features. We used a sparse GP to deal with the high dimensionality of the data.

DL I, RoNIN. Yan et al. [29] published RoNIN, the current state-of-the-art in velocity estimation of homogeneous walking in publicly available datasets. RoNIN modifies and extends ResNet [65], see Section 3.2. It replaces all type II units by type I units; replaces the Softmax and classification layers by a fully connected layer and a (half-mean-squared) regression layer; and, adapts the input dimension to a six-dimensional input vector with a length of 200 that covers 1.200 raw measurements (=6 × 200) for both 3D acc and 3D gyr at a sampling rate of 100 Hz. In preliminary work, we applied the original RoNIN code with its standard configuration to our datasets. However, to get to the best possible RoNIN-type DL-based estimator, we conducted a grid search among possible variations and configurations (input combinations of 1D and 2D SMVs (of acc and gyr), different window sizes of the input vectors (variations from 64 to 1024 Hz), and a combination of type I and type II residual units). With an input window size of 256 measurements on 2D SMVs (of acc and gyr) and with the other ideal configuration parameters, see Appendix A.3 for details, the result is the best RoNIN-type DL-based method for our dataset. This is what Section 5 uses in the comparison.

DL II, C/RNN. In previous work [32], we proposed a DL-based model that employs a shallow CNN and a BLSTM that performs well on our dataset. The key aspects of this C/RNN are: 1D inertial data input sequence (with m = 128 measurements per input window), a first convolution layer (128 filter kernels, each of size 1 × 3), followed by a BN layer, and a ReLU, a second convolution layer (128 filter kernels of size 1 × 3), followed by a BN and a ReLU, a flattening layer, a BLSTM layer (=2 × LSTM layers), a dropout layer, a fully connected layer (provides 1 × 1 output), and a final regression layer that calculates the loss with a half-mean-squared error. The architecture was also optimized on our dataset in a grid search, see Appendix A.4 for the results.

DL III, Hybrid. To find the optimal architecture and its parameters for our hybrid method, we also performed a grid search, see Appendix A.5 for details, which evaluated different architectures, such as LSTM, BLSTM, ResNet, and combinations of them. In the search, we kept all LSTM and BLSTM blocks identically w.r.t. the number of hidden layers and the number of cells. ResNet-18 [65] was adjusted to our input and output dimensions. We tested dropout layers between or after the BLSTM layers (after was better, with an ideal dropout rate of 50%). We trained each set of parameters for 100 epochs, with early stopping based on the validation set performance to prevent over-fitting. The resulting best combination of ResNet-18 and two BLSTM blocks (each with 128 LSTM cells) that we have described in Section 3.2 yields the best accuracy at short inference times. The parameters used in Figure 4 were also found in the grid search to work best. As its final stage our design has an LKF, we parameterize it based on the predictions $v^{\prime }$ of the hybrid method, as follows. We train the LKF’s covariance of the measurement noise on the predictions $v^{\prime }$ and also optimize the covariance of the state transition noise until the LKF provides a robust and plausible performance on $v^{\prime }$, and can thus predict optimized velocities $v^{\prime \prime }$ on unknown test data. For completeness and fairness, we also apply LKF to the $v^{\prime }$ estimates of all other methods to assess the effects of an LKF on the accuracy of each method. We provide details on the impact of an LKF on the results of each method in Section 5.1.

## 5. Benchmark Results

As publicly available datasets, such as the OxIOD data set [59], the RIDI data set [31], or the RoNIN data set [29], do not cover the highly dynamic motions that we want to track, we could not use them for our experiments. Our benchmarks compare the methods along three dimensions: velocity estimation accuracy (Section 5.1), computational effort (Section 5.2), and generalizability (Section 5.3) to unknown data. Each of these criteria need different sub-sets of data, see Table 1. First of all, we split the pool of 23 subjects into three groups. Twenty-two users provide data from the same type of inertial sensors. One user provides data from two special inertial sensors and we use his/her “unknown devices” data in the generalizability benchmark. From the 22 users, we use data from randomly selected 20 in the accuracy benchmark (Section 5.1) and the computational effort benchmark (Section 5.2). We used data from the remaining two users (so called left-outs) in the generalizability benchmarks (Section 5.3). For all three benchmarks, we always use the best-performing estimator configurations. For PDR, we achieved this by training on V2. The data-driven methods performed best when trained on V3.

### 5.1. Accuracy

Dataset Statistics. The benchmark works with data from the 20 randomly selected subjects. To explicitly investigate the accuracy of the velocity estimation, it evaluates all five methods on known training data (70% = 14 subjects), quasi-unknown validation data (10% = 2 subjects), and unknown test data (20% = 4 subjects). The left-outs were at no time part of the benchmark. The data from the 20 subjects were pre-processed with the optimal parameter configuration ($N_{w}$ = 128 and $F_{s}$ = 100 Hz), as described in Section 3.1 and Section 4.2 to yield the segment, window, or feature bundles (and their variants), each split into 70/10/20. For detailed dataset statistics and sizes, see the upper part of Table 1. Note that the total distance traveled for both sensors and the total number of windows (214 km and 129,375) in Section 4.2 reflect all 23 subjects. Table 1 shows values for the number of subjects picked for a sub-set. For example, V1 has 160 segments or 112,500 windows, or feature bundles, because it holds data of 20 users × two sensors × 30 min × 60 s × 100 Hz sampling rate 64 Hz per 50% overlapping window size ($N_{w}/2$ = 128/2 = 64) for four activities.

**Figure 9:**
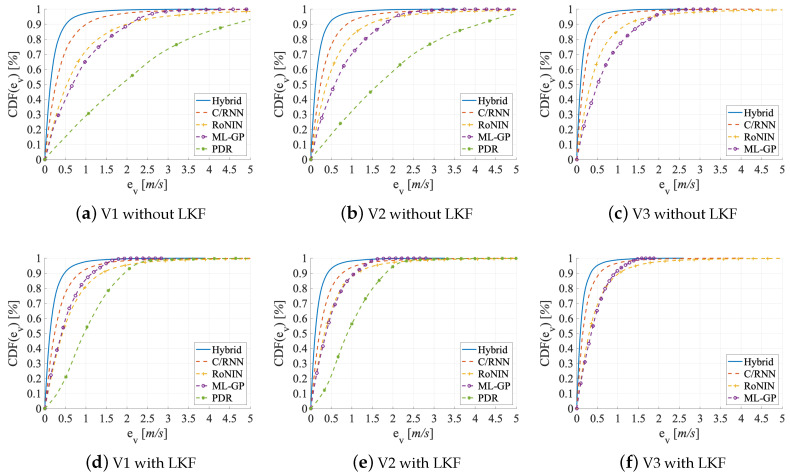
CDF on test sets V1–V3, both without and with linear Kalman filter (LKF).

**Figure 10:**
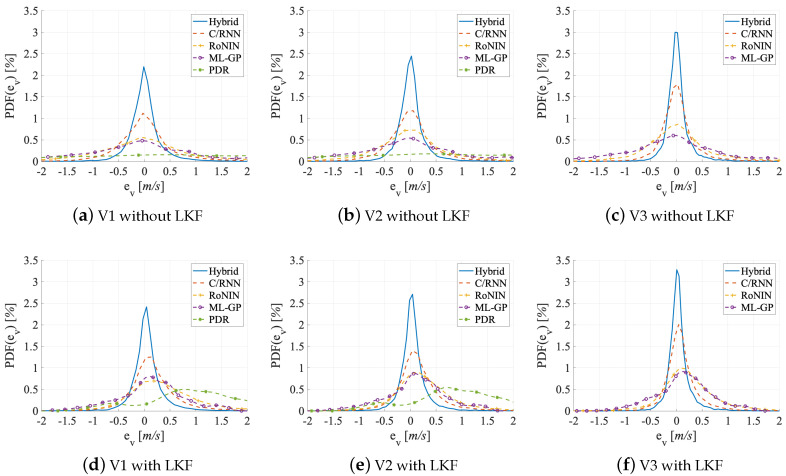
PDF on test sets V1–V3, both without and with LKF.

**Figure 11:**
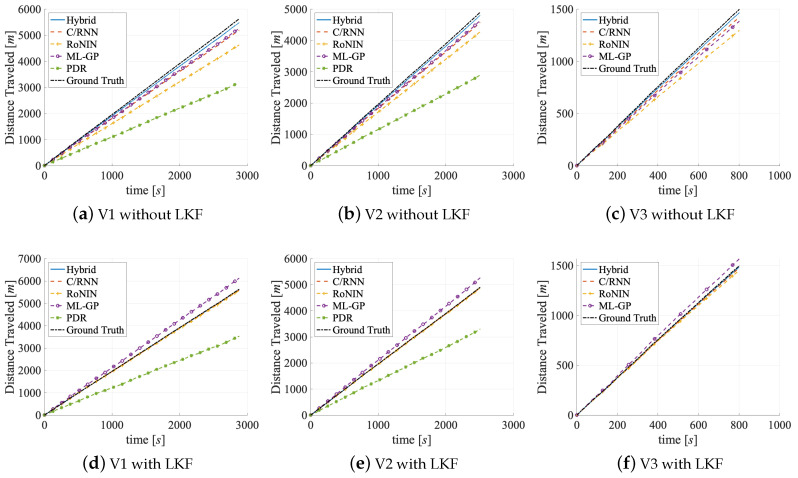
Distance error total (DET) over time on the test sets V1–V3, both without and with LKF.

Metrics. The accuracy of a velocity estimator is given by (1) the error of the velocity and (2) the error of the distance traveled. For the former we use four error variables and two error functions: the mean absolute error (MAE), the mean square error (MSE), the root mean square error (RMSE), and the circular error probability (CEP). To gauge the error of the traveled distance (2) we use two error variables: the distance error per meter (DEPM) and the distance error total (DET). We determine all errors on the estimated velocity $v^{\prime }$, the optimized velocity $v^{\prime \prime }$, and the ground truth velocity $v_{ref}$, respectively, over time for the distance errors. By default, Hybrid uses an LKF as its final stage. Because adding an LKF also improves the results of the other estimators, we also show LKF-free evaluation numbers and graphs for Hybrid in Table 2 and Figure 9, Figure 10 and Figure 11.

Evaluation on V1. V1 contains activities that vary in their duration between users. Therefore, the estimators have to deal with redundant information (since neither the activities nor the velocities are equally distributed). Table 2 holds the results in its upper group of rows. On the test set, Hybrid performs best (MAE = 0.25 m/s), followed by C/RNN (MAE = 0.47 m/s). RoNIN (MAE = 0.88 m/s) and ML-GP (MAE = 0.89 m/s) are on par. Likewise, the CDF-Figure 9a shows that all four data-driven models provide $v^{\prime }$ estimates with an error below 1.25 m in 70% of all cases. PDR (with $K_{g}$) yields the worst MAE (=2.16 m/s). Looking at the robustness of the methods to small and large outliers Hybrid (best) has fewer large outliers (RMSE = 0.40 m/s), followed by C/RNN (RMSE = 0.77 m/s), while RoNIN (RMSE = 1.46 m/s) and ML-GP (RMSE = 1.17 m/s) have more serious problems. PDR has the largest outliers (RMSE = 2.71 m/s). These are very likely due to model inconsistencies that are introduced by the variety of motions. Interestingly, ML-GP (MSE = 1.38 m/s) seems to handle outliers a little better than RoNIN (MSE = 2.15 m/s). This might be due to RoNIN’s mapping of measurements to a single fingerprint number. The PDF-Figure 10a supports these findings and underlines the robustness of C/RNN and Hybrid, as they form more narrow and higher amplitudes (around zero) than the other estimators.

The distance errors show that PDR has serious problems (DET = 2.1 km), followed by RoNIN (DET = 460.47 m), while the other three methods do not show abnormalities. In the DET-Figure 11a, both PDR and RoNIN are farther away from the ground truth than the other three methods. Interestingly, while PDR makes large DET mistakes and also has a large DEPM ( = 0.37 m), for RoNIN its high DET value does not correlate with its low DEPM value. Obviously, DEPM does not necessarily directly correlate with DEPM, instead the large RMSE values (PDR: 2.71 m/s; RoNIN: 1.46 m/s) suggest that there are outliers that may cause the high DET values.

Looking at the effects of $f_{s}$ and $f_{t}$ on the accuracy, we believe that ML-GP surpasses RoNIN, as it represents the reliability of the represented $f_{s}$. A comparison of the ML-GP curve with the RoNIN curve at $e_{v}$>2 m/s in the CDF-Figure 9a supports the lower DET values of ML-GP. The shapes of the error PDFs (Figure 10) are mostly symmetric and almost Gaussian in shape. However, a noticeable effect of systematic overestimation is present for the PDR errors when they are filtered with the LKF. We think that RoNIN may overfit too much to the redundant data, while ML-GP processes them effectively and is less susceptible to unequally distributed data. In contrast, the C/RNN and Hybrid memory cells may contribute to remembering what knowledge $f_{t}$ to store and what to forget. Here, tracing the emergence of $f_{s}$ over time may add to the accuracy of $f_{t}$. However, C/RNN is significantly worse than Hybrid across all metrics (C/RNN: $CEP_{95}$ = 1.49 m/s versus Hybrid: $CEP_{95}$ = 0.77 m/s). This might indicate that the deeper feature extractor (ResNet) of Hybrid performs better than the shallow one of C/RNN (2 × convolution layers), as the rest of their architectures is similar. The RMSE numbers also are an early indication that time and context $f_{t}$ appear to have a significantly positive impact on accuracy: C/RNN and Hybrid that both exploit $f_{s}$ and $f_{t}$ lead to much lower errors (RMSE = 0.77 m/s and 0.40 m/s) than ML-GP and RoNIN, which only exploit $f_{s}$ (RMSE = 1.17 m/s and 1.46 m/s). This is in line with the CDF-Figure 9a that shows that, in 90% of all cases, the data-driven methods that take $f_{t}$ into account provide errors below 1 m, clearly outperforming the other methods. A pre-study of PDR showed that, even with subject- and activity-specific coefficients $K_{sa}$, the errors remain high (MAE = 1.97 m/s, RMSE = 2.41 m/s, and DEPM = 0.31 m). While, with $K_{g}$, PDR has a high distance error DET = 2.103 m, with $K_{sa}$ it only improves to DET = 1.73 m. Only a $K_{sa}$ together with a fine-tuning of the step detection (we optimized a threshold for the peak detector on a specific test segment) yields lower errors (MAE = 1.13 m/s, RMSE = 1.87 m/s, DEPM = 0.27 m/s). However, even with an elaborate and impractical optimization that pre-classifies an activity in the sensor stream and selects a suitable subject-activity-specific $K_{sa}$, PDR performs worse than the others

Evaluation on V2. In V2, activities are pruned to have the same duration. Table 2 shows the results in its middle group of rows. RoNIN benefits more than ML-GP when moving from V1 to V2 (MAE: −0.24 m/s vs. −0.21 m/s and $CEP_{95}$: −0.79 m/s vs. −0.32 m/s). The reason might be that RoNIN is no longer forced to memorize or overfit to redundant data (e.g., some users move much slower than others, even during fast activities, such as running, V2 adjusts this imbalance slightly and the redundancy of slow velocity data is slightly reduced), while ML-GP does no longer profit from its functional structure. For C/RNN and Hybrid there are only mild improvements of accuracy (MAE: around −4 cm/s and $CEP_{95}$: around −12 cm/s). We think that this is because they already track only the most important features. A comparison of the curves in the CDF-Figure 9a,b reveals that they are all pushed towards the upper left. All four data-driven methods now show errors that are below 1 m/s in 70% of all cases as compared to 1.25 m in 70% on V1. As with V1, PDR (with $K_{g}$) still performs worse (DET) than the four data-driven methods, even though there is a significant gain when moving from V1 to V2 (MAE: −0.23 m/s and $CEP_{95}$: −0.56 m/s).

Looking at the small and large outliers of the methods, we see that moving from V1 to V2 remarkably reduces the number of large outliers as MSE and RMSE both decrease across all five estimators by up to 46% (MSE of RoNIN). However, a closer look shows that, while Hybrid has the fewest outliers (MSE = 0.12 m/s; RMSE = 0.32 m/s), followed by C/RNN (MSE = 0.48 m/s; RMSE = 0.69 m/s), the other estimators still have serious problems (ML-GP: MSE = 1.04 m/s; RMSE = 1.02 m/s; RoNIN: MSE = 1.15 m/s; RMSE = 1.07 m/s). This is in line with the more narrow bell shape of all curves, except PDR’s curve, in the PDF-Figure 10b. The smaller MSE and RMSE values on V2 than on V1 might indicate that all methods focus more on denoising (i.e., filtering out small and large outliers) than on learning redundant information.

The distance errors have an impact on the robustness. For PDR, the DET shrinks significantly from 2.1 km to 1.8 km. DET also shrinks for RoNIN by a remarkable 250 m, which is in line with its RMSE reduction by about 40 cm. ML-GP, C/RNN, and Hybrid also show lower errors across all metrics, but they do not benefit as much as ML-GP and RoNIN. The comparison of the PDF-Figure 10a, b shows a noticeable increase in the amplitudes of RoNIN, C/RNN, and Hybrid that also form a more narrow bell shape. RoNIN’s handling of small outliers is now closer to ML-GP (their MSE differs by 0.11 m/s on V2 vs. 0.77 m/s on V1). A decrease in the DEPM values (ML-GP: 0.014 m vs. RoNIN: 0.008 m) might cause the ML-GP curve to be closer to the ground truth than RoNIN’s curve, see the DET-Figure 11b. This might also indicate that RoNIN now has less handicap to both separate and memorize important information.

While we do not see a significant benefit of the $f_{t}$ tracking methods (C/RNN and Hybrid), the methods that track $f_{s}$ benefit the most from working on a dataset with less redundancy.

Evaluation on V3. Remember that we do not evaluate PDR on V3, as there are no segments. In V3, velocities are homogeneously distributed over 3 to 13 km/h for all users; there is less redundancy than in V2. Table 2 holds the results in its bottom rows. While ML-GP (MAE: −16% and $CEP_{95}$: −16%) and RoNIN (MAE: −37% and $CEP_{95}$: −38%) benefited the most when moving from V1 to V2, now C/RNN (MAE: −50% and $CEP_{95}$: −49%) and Hybrid (MAE: −19% and $CEP_{95}$: −22%), see the strongest improvements at a much more fine-grained level (below 20 cm) when moving from V2 to V3. Hybrid (best) shows the smallest error on V3 (MAE = 0.17 m/s; $CEP_{95}$ = 0.53 m/s), followed by C/RNN (MAE = 0.28 m/s; $CEP_{95}$ = 0.89 m/s). Of course, all four methods in the accuracy benchmark perform remarkably better with V3 than on the other data set variants. The reason might be that V3 has a suitable distribution of data and therefore also statistical information. It is known that such an information distribution may lead to quasi optimal results of the estimators [77]. This is in line with the CDF-Figure 9c that now tells us that, in 70% of all cases, the errors of the four data-driven methods are below 0.75 m. Now, all methods probably focus on denoising the raw data, extracting the best $f_{s}$ and $f_{t}$ features, and having a clear sight on what is important to memorize.

Looking at how the methods handle small and large outliers on V3, we can see the same trend as for MAE and $CEP_{95}$. For all four methods, both the MSE and RMSE values significantly decrease when moving from V2 to V3. C/RNN benefits the most when moving from V2 to V3 (MSE: −56%; RMSE: −30%), followed by Hybrid (MSE: −30%, RMSE: −19%) and RoNIN (MSE: −26%; RMSE: −14%). C/RNN and Hybrid also have the fewest outliers (MSE = 0.21 m vs 0.07 m and RMSE = 0.46 m vs. 0.27 m). ML-GP benefits the least when moving from V2 to V3 (MSE: −25%; RMSE: −14%). On V3, there seems to be the least noise in the results. All of the methods seem to efficiently denoise the data.

The distance errors and their impact on the robustness benefit from moving to V3 in the same way as the outliers. When compared to V1 and V2, all four methods achieve their best DET values on V3. As C/RNN and Hybrid yield the lowest DET values at a total distance of 1.5 km, their curves get closest to the ground truth, see DET-Figure 11c.

As we have seen on the V2 dataset, both C/RNN and Hybrid (which use both $f_{s}$ and $f_{t}$) achieve much smaller errors across all metrics than ML-GP and RoNIN (which only use $f_{s}$). Thus, we think that the ability of a BLSTM in combination with a dropout layer (C/RNN and Hybrid) to track $f_{t}$ in a window appears to have a significantly positive impact on the accuracy.

Added Value of the Post-Processing with an LKF. The final LKF does enhance the accuracy of all five methods on datasets V1 to V3. The results are in the grey lines of Table 2. The validation dataset does not include the LKF, as we use it to train the LKF on the predictions of each method.

There are three key findings. First, the post-processing with an LKF significantly increases the velocity and the distance estimation accuracy of all methods on all datasets, on average by: V1: 145%, V2: 70%, and V3: 60%. Compare the black lines to the corresponding grey lines in Table 2. Second, methods that only work on $f_{s}$ (PDR, ML-GP, and RoNIN) benefit more from the LKF than C/RNN and Hybrid. On V1, the average improvement of MAE, MSE, RMSE, and $CEP_{95}$ for the former is PDR: −185%, ML-GP: −108%, RoNIN: −67% versus for the latter C/RNN: −30%, and Hybrid: −14%. Third, an added LKF lowers the DET for all methods (except for ML-GP) by approximately 50%.

On both V1 and V2, an added LKF helps PDR the most (MAE: −1 m; $CEP_{95}$: −3 m). The PDR curve changes the most between the CDF-Figure 9d,e. The added LKF shows a better denoising and interpolation for the methods that work with $f_{s}$ (ML-GP and RoNIN) than for the two $f_{t}$ tracking methods (C/RNN and Hybrid). The CDF-Figure 9d–f underline this: all of the curves are pushed towards the upper left (ML-GP even surpasses RoNIN), so that, with an applied LKF, the errors get below 0.5 m in 70% of all V3 cases, which is a 125% improvement.

With respect to small and large outliers, we see the same trend of MSE and RMSE as with MAE and $CEP_{95}$. While the LKF removes outliers for all estimators, PDR profits most (MSE: −5.8 m/s, RMSE: −1.25 m/s), followed by ML-GP (MSE: −0.94 m/s, RMSE: −0.51 m/s) and RoNIN (MSE: −1.22 m/s, RMSE: −0.49 m/s). We suppose that the LKF use the validation data to track the weak spots of these characterizations and improve the denoising and, hence, the MSE and RMSE values, see the PDF-Figure 10d–f that also shows that the effect is the strongest on V3.

Looking at the distance errors, the DET-Figure 11d–f shows that with an LKF all curves, except ML-GP, get much closer to the ground truth. Other than that, the effect of the LKF on the methods’ DEPM and DET values are a bit blurry. For three methods, the LKF even downgrades the average DEPM (PDR: +17%, RoNIN: +86%, and C/RNN: +59%), perhaps because it fails to spot and track some outliers. On the other hand, although the LKF reduces the DEPM value of ML-GP, ML-GP has the worst DET values across all datasets (average DET: 163 m). This is in line with ML-GP’s smaller reduction of RMSE values. The other four methods benefit from the LKF and show improved DET values (PDR: −724 m; RoNIN: −209 m; C/RNN: −43 m, and Hybrid: −8 m).

Regardless of whether methods work with $f_{s}$ or with $f_{t}$, they all benefit from the LKF. The LKF has a stronger effect on the larger errors of the $f_{s}$ methods, perhaps because the $f_{t}$ methods (C/RNN and Hybrid) already consider the time as they learn a state-space model that the LKF also provides.

### 5.2. Computational Effort

We measure the training and inference times of of all five methods on the same sub-set of V1 that is used in the accuracy benchmark. We measure the training times on the training set and the inference times on the test set. For Hybrid, we also measure the training times of the post-processing step on the validation set. The training and the inference times both highly depend on parameters of the method, e.g., early stopping with patience, width, depth, and cell type of a NN.

Setup. For the benchmarks we use off-the-shelf hardware components. There are two setups: (1) one core of a single CPU (Intel Core i7 4 × 3.6 GHz), (2) a machine equipped with a single GPU (NVIDIA GTX1080TI 11GB). For PDR, ML-GP, and Hybrid’s LKF, there are no GPU implementations. Hence, for PDR and ML-GP, we only have measured on setup (1). For the other systems, we have CPU-only measurements (setup (1)) and also measurements taken on setup (2). Here, for RoNIN and C/RNN, the CPU core only coordinates the GPU implementations. For Hybrid it also runs the LKF. We separately measure the run-times of the full training (all windows/features) and the inference for a single input sequence (below we use the term live). While data pre-processing times are negligible for training (as we only do it once but run iteratively on the data), we included them in them for live, as they matter here for the real-life application of the methods.

Evaluation Design: for the dataset V1, Table 3 lists $t_{train}$ (for all windows/features or segments) and $t_{live}$ (average time that it takes to predict the velocity on a single window/feature set). As Hybrid uses an LKF, its total training time is $t_{train}$ = $t_{train}^{pure}$+$t_{train}^{LKF}$, where $t_{train}^{pure}$ covers ResNet and BLSTM on the complete training set (on all the 112,500 windows) and $t_{train}^{LKF}$ shows how long the trained Hybrid model needs to predict $v^{\prime }$ on the full validation set (11,250 windows) plus how long it takes to train the LKF on these predictions $v^{\prime }$ (11,250 $\times v^{\prime }$). Hybrid’s live time is $t_{live}$ = $t_{live}^{pure}$+$t_{live}^{LKF}$ with $t_{live}^{pure}$ averaging how long the trained pure Hybrid takes to predict $v^{\prime }$ for each of the 22,500 windows of the test dataset and $t_{live}^{LKF}$ averaging how long the trained LKF takes to predict $v^{\prime \prime }$.

For the training, the GPU times are faster than the CPU times by a linear factor, see Table 3. For the live time, the GPU times are slower than the CPU times, as the GPU cannot use its computing power on a single window. PDR is by far the fastest method for the training ($t_{train}$ = 0.2 h). Due to their gradient optimization, the other (data-driven) methods take more than 34× longer. ML-GP trains the slowest ($t_{train}$ = 23.6 h), which is in line with Liu et al. [83]. RoNIN takes long ($t_{train}$ = 11.2 h), as it builds upon ResNet that is known to be computationally demanding because of its many parameters [67]. As C/RNN uses a flat CNN and, thus, employs fewer parameters, it is faster ($t_{train}$ = 6.9 h). For Hybrid, the extra BLSTM layers (≈3.4 h) and the LKF ($t_{train}^{LKF}$ = 0.98 h) slow down the training times ($t_{train}$ = 15.6 h). With respect to the live times in Hz, again PDR is by far the fastest method ($t_{live}$ = 1370 Hz). This time, ML-GP is about 3× faster than the other data-driven methods. For both C/RNN and Hybrid, the BLSTM layers slow down the prediction by about 40 Hz. The LKF only adds about 0.002 s per window. Thus, it is clearly affordable to add an LKF to any of the estimators.

### 5.3. Generalizability

**Figure 12:**
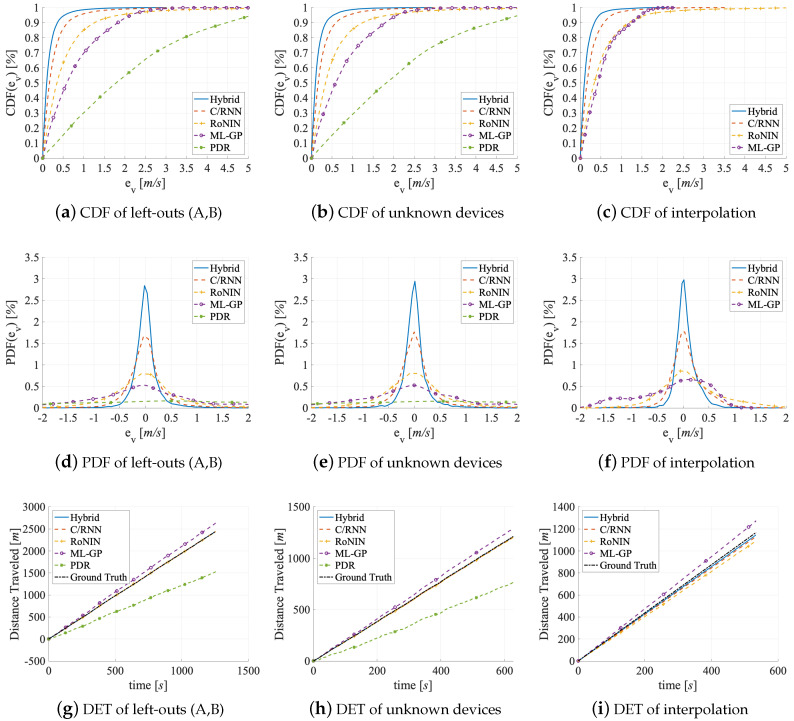
CDF, PDF, and DET of the generalizability benchmarks: left-outs (A,B), unknown devices, and the interpolation datasets

There are four experiments in this benchmark, see the last group of rows in Table 1. To investigate the generalizability of the five methods on unknown data, we look at their performance on (1) the unknown data of the Left-Outs, (2) of the user with two special sensors of Unknown Devices, (3) Interpolation between two ranges of velocities, and (4) test the methods’ Extrapolation abilities. Before the Left-Outs and Unknown Devices experiments, all methods were trained in the best possible way (PDR on V2, others on V3). Only Hybrid uses an LKF. Before the Interpolation and Extrapolation experiments, we have to train the four data-driven methods from scratch. We use the V3 data of the 20 subjects for this (without the left-outs and the special devices user). The two reasons for this choice are the comparability to the other benchmarks and the fact that the data-driven methods perform better on V3 than on V1 and V2. For the four experiments, Table 4 lists the six velocity error metrics known from the accuracy benchmark. We also visualize the errors as: CDF, PDF, and DT graphs in Figure 12.

Left-Outs. The two left-out users moved fairly similarly: left-out A moves at 0.74 m/s on average (min.: 2.5 m/s, max.: 3,9 m/s, SD: 0.41 m/s). Left-out B, who indicated that he does not exercise as often as A, moves somewhat slower at 0.66 m/s on average (min.: 2.4 m/s, max.: 3.6 m/s, SD: 0.38 m/s). Table 5 holds the errors for the two left-out users per activity. We evaluated the estimators on the left-out data both individually and in combination. The main take-aways are: (1) all estimators generalize well (small error readings) at least if unknown users move at velocities that are covered by the training data. They also generalize robustly (similar lines for A, B, and A+B in Table 4). (2) Despite the slightly different velocity of the two left-outs the generalization is similar. All of the estimators only make small errors across all metrics (that are also on the level of the test set of V3 (V2 for PDR) in the accuracy benchmark, see Table 2).

Similar to the accuracy benchmark, PDR generalizes worst, where the Hybrid generalizes the best. All of the methods handle left-out B a bit better than A, across all error metrics, probably because the training data does only partially cover A’s velocity. The results for the combined left-outs A+B in Table 4 are similar to those for the individual left-outs, but the estimators score slightly worse across all error metrics (about −8%) than on the test dataset of V3 (V2 for PDR). The scores are still impressive, but the slight deviation might be caused by missing motion patterns and missing velocities in the training data. PDR struggles with the left-outs A+B, since its parameterization does not cover the kinetics of their movements.

Hybrid and C/RNN are the most robust estimators, as they show the highest peaks with the most narrow amplitudes in Figure 12d. However, their MSE and RMSE values are worse (by −11%) than their results in the accuracy benchmark, see Table 2. We explain this with the unknown and highly individual motion patterns between windows that change over time. Even the $f_{t}$ tracking methods struggle slightly when they work with the faster motions of left-out A that are not covered by the training data. PDR, ML-GP, and RoNIN suffer even more from this.

Interestingly, the DET values for PDR (DET = 914 m) and ML-GP (DET = 188 m) are almost the sum of the individual DET values for A and B, while the DEPM values in all three lines of Table 4 are the same. We assume that PDR’s accumulation and underestimation of velocity is due to undetected steps from left-out A, as $K_{g}$ may not match his faster movement patterns. Instead, the accumulation of DET values by ML-GP and the overshooting of the velocities may indicate that the velocity ranges of the training data cover mostly faster velocities. This bias to higher velocities can explain the slightly higher error numbers. Instead, the DL-based methods (DET<5 m on average) do not show an accumulation of DET values. They all get close to the ground truth, probably because they implicitly learn to handle more complex feature dependencies directly from the data.

Unknown Devices. In this experiment, two special sensors (left and right pocket of one left-out user) move at an average velocity of 2.6 m/s (min.: 1.3 m/s, max.: 3.5 m/s, SD: 0.28 m/s). Table 4 holds the errors in the second group of rows. The main take-away is that all estimators generalize well (error readings slightly worse than the test set of V3 (V2 for PDR) in the accuracy benchmark, see Table 2) if known sensors move at velocities that are covered by the training data.

All five estimators generalize slightly worse (PDR worst, Hybrid best) across all error metrics for the unknown devices than for the left-outs. Additionally, their accuracy on the unknown devices is also slightly worse than on V3 (V2 for PDR), but comparable to their results on V1, see Table 2. This can be attributed to the fact that the methods not only struggle with unknown movement patterns, but also with unknown scaling of the sensor measurements or unknown short- and long-term noise.

The robustness of all methods against outliers is similar for the unknown devices and for the left-outs, see Figure 12e,d. The denoising of Hybrid and C/RNN is more robust against both outliers and variations in the scaling of the sensor readings. All of the methods show worse MSE and RMSE values (by −15%) than they did in the accuracy benchmark, see Table 2. As these results are in line with the results for the left-outs, both the highly individual motion patterns of the unknown user that carries the two sensor as well as the unknown sensitivity to motion of the unknown sensors may cause the higher MSE and RMSE errors. As different scaling of the sensor readings directly convert to different amplitudes and the methods interpret these as different velocities, there are larger outliers.

In line with their higher MSE and RMSE values, the distance errors of PDR (DET = 506 m) and ML-GP (DET = 101 m) are the worst, see Figure 12h. This is plausible as their bad MSE and RMSE values (that may stem from a different scaling of the sensor data) directly convert to higher DET values. Again, PDR under- and ML-GP overshoots the ground truth, see Figure 12h. The reason for PDR’s failure to detect steps is that the different scaling no longer matches the thresholds of the peak detector that was trained on other sensors with a different scaling. Instead, the DL-based methods (DET<12 m) are mapped well to the ground truth.

Interpolation. We let the methods interpolate between two ranges of velocities. We train with windows (or features) of slow ($v_{ref}\in$[3,7) km/h) and fast ($v_{ref}\in$(9,13] km/h) velocities. Recall that V3 hold these velocities in four of its five 2 km/h range bins. To gauge the interpolation abilities, we use the remaining range bin with normal velocities ($v_{ref}\in$[7,9] km/h) and split this data into validation (33% = 2.059 windows/features) and test datasets (67% = 4.181 windows/features). We train the four estimators with windows (or features) of slow ($v_{ref}\in$[3,7) km/h) and fast ($v_{ref}\in$(9,13] km/h) velocities. We evaluate them on normal velocities ($v_{ref}\in$(7,9) km/h). The results are in rows at the bottom of Table 4. Remember that we cannot evaluate PDR on V3. The main take-away is that all four data-driven methods are able to interpolate between two distinct ranges of velocities and provide velocity estimates at a similar or even higher accuracy than they did on the test set of V3 (see Table 2). The four estimators perform similar on the validation and test datasets across all error metrics.

While ML-GP (worst) and RoNIN perform similar (MAE = 0.5, $CEP_{95}$ = 1.5 [m/s]), C/RNN and Hybrid (best) perform slightly better (MAE = 0.26, $CEP_{95}$ = 0.76 vs MAE = 0.16, $CEP_{95}$ = 0.44 [m/s]). Figure 12c reveals that, in 70% of cases, all four methods show errors below 0.7 m/s. The graph also shows that ML-GP clearly outperforms RoNIN above probabilities of 0.9. Our explanation is that ML-GP learns to align its internal distributions, such that they cover the unknown range of velocities very well, while RoNIN cannot make that much use of its fingerprint learning mechanism. The good performance of C/RNN and Hybrid is in line with all other findings, where the exploitation of $f_{t}$ almost always outperforms the use of just $f_{s}$. Both of the estimators benefit from using an optimal training set that also covers the borderline velocities. The four methods interpolate about +3% better than they estimate in the accuracy benchmark (Table 2). Here it helps that the unknown range of velocities is comparably small, so that only short-range interpolations are needed.

The robustness of all methods is similar to the accuracy benchmark, as the training and test data does not change (same 20 users), even though we left out other parts here. The DEPM and the DET both scale linear with the MSE and RMSE. Again, ML-GP is the only method that overshoots the ground truth, see Figure 12i, as the velocity ranges are slightly positively imbalanced within each velocity range/bin. While RoNIN seems to handle such imbalances better than ML-GP, it undershoots and yields slightly lower errors across all metrics. C/RNN and Hybrid interpolate slightly better (DET = 36 m vs. DET = 21 m) than the methods that only track $f_{s}$. However, we cannot reason how their time and context sensitivity impacts the interpolation. We guess that the $f_{t}$ features force the C/RNN and Hybrid models to implicitly focus more on the connectivity and inter-dependencies of velocities.

Extrapolation. To test the methods’ extrapolation abilities, the training data now contain slow to normal velocities ($v_{ref}\in$[3,11) km/h), i.e., the first four range bins. The evaluation data is the remaining range bin of fast velocities ($v_{ref}\in$(9,13) km/h). Again, we split this data into validation (33% = 2.059 windows/features) and test datasets (67% = 4.181 windows/features). Note that for simplicity we just reuse and rearrange the five bins of V3, each with 6240 windows of 20 users × two sensors × 156 windows × one per bin (bin size of 2 km/h), see Section 4.2 for details. Remember that we cannot evaluate PDR on V3. There are neither numbers in Table 4 nor graphs, as all methods are unable to extrapolate well.

All of the estimators show high errors (5–10× compared to the accuracy benchmark) on both the validation and test data that also fluctuate randomly. There is no clear relation between the input and the extrapolation. As data-driven methods learn to fit the validation data, they can only work well on the test data if both of the datasets stem from the same data pool. However, since, in this experiment, all methods fail on both the validation and the test sets, this indicates that they really cannot extrapolate. The closer the velocities are to the training data (i.e., closer to 11 km/h) the better the accuracy gets. It decreases when testing on data that exceeds the maximal velocities covered by the training data. Hence, we cannot recommend using the methods for extrapolation for velocities that are not covered in the training data.

Because none of the methods extrapolate sufficiently well on specially selected velocities, we tested their ability to extrapolate to random motion patterns. To do so, we only trained on walking, jogging, and running data, and left out the random activities. Because the results do not show anomalies, we only discuss the traveled distance error: the PDR method again overshoots significantly by 759 m, while ML-GP (−200 m), RoNIN (−142 m), C/RNN (−87 m), and Hybrid (−16 m) undershoot. Our explanation is that the data from walking, jogging, and running already contain motion patterns from users that moved at varying motions with abrupt direction changes and, hence, all of the methods do learn random activity features. Hence, we recommend using the data-driven methods for extrapolation when the motion patterns are covered in the training data.

### 5.4. Effects of Windows Size

Typically, the velocity estimators propagate errors along the state-space, as they continuously integrate errors over time. We break this cycle by sliding quasi-independent windows over the signal [66,84]. However, the window size $N_{w}$ and the window overlap are parameters that affect the model with respect to both acceleration and angular rate. The window size also affects the learning rate. Brownlee et al. [85] found that, in general, the maximal input sequence is $N_{w}$ = 400 (at 100 Hz) for LSTM cells to work fail-safe. Our model seems to learn long-term dependencies $f_{t}$ of measurements within windows already from 128 samples (1.28 s) and provided the highest accuracy for all methods. For completeness, here we sketch the performance fluctuations that are caused by changing $N_{w}$.

While the inference times $t_{live}$ are not affected (SD ±0.002 s) by $N_{w}$ and by the overlap, we see effects for the training times $t_{train}$. A window size $N_{w}$ = 512>128 results in redundant information, slows down the training process for data-driven methods ($t_{train}$ +7.3h at 100 Hz), lowers valuable information content ($f_{s}$) for ML-GP (as the features blur out) and RoNIN (test set of V1: RMSE 1.01 m/s and 1.16 m/s), does not help C/RNN and Hybrid (test set of V1: RMSE 0.74 m/s and 0.61 m/s), and raises the computational effort, even for PDR methods. $N_{w}$ = 64 < 128 increases the computational performance ($t_{train}$−4.1h at 100 Hz), but yields remarkably less accurate estimates on the test set of V1 (RMSE [ m/s]: ML-GP 1.94, RoNIN 1.92, C/RNN 1.89, Hybrid 1.78, and compare line V1 of Table 2). Our experiments revealed that all four data-driven methods perform similarly on both 1D- and 2D-SMVs (RMSE SD ≤ 0.06 m/s), but 1D-SMV saves computational costs ($t_{train}$−2.9h at 100 Hz). A low $N_{w}$ may still be an option for smartphone-based localization, when lower energy consumption is important.

## 6. Conclusions and Future Work

This article proposed a novel Hybrid velocity estimator that combines DL with a Bayesian filter on rotation-invariant signal streams. Its combination of convolutional (ResNet-18) and BLSTM NNs extracts spatial features from the sensor signals and tracks their temporal relationships to estimate velocities. An LKF further optimizes theses estimates. We compared our approach to state-of-the-art methods such as PDR, ML-GP (Gaussian Processes Regression based on handcrafted features), RoNIN (ResNet), C/RNN (CNN+BLSTM) and showed that we outperform state of the art in terms of accuracy, generalizability, and computational cost. We also proved that an additional post-processing with a linear Kalman filter further improves the results.

Future work examines how training on synthetic data and retraining on real data may help to push the boundaries of generality. We will also explicitly examine other types of movements, such as jumping and climbing stairs, and also examine other natural positions of the IMU, such as head and hands. We will also combine Hybrid’s velocity estimates with a PDR that enables multi-sensor fusion (based on low-frequency radio-based positions and IMU-based orientations) in a single network and compare them to well-designed state-of-the-art PDR methods.

## Figures and Tables

**Table 1:** Dataset statistics.

Name	Num. of Users [#]	Total [#]		Training [#]		Validation [#]		Testing [#]		Duration	Distance	$v_{\mathrm{ref}}$ [m/s]		
		Segm.	Wind./Feat.	Segm.	Wind./Feat.	Segm.	Wind./Feat.	Segm.	Wind./Feat.	[min]	[km]	*avg.*	min	*max*
**Accuracy**														
V1	20	160	112.500	112	78.750	16	11.250	32	22.500	1.203	185.40	2.5	0.8	7.9
V2	20	160	97.950	112	68.565	16	9.795	32	19.590	1.045	161.42	2.6	0.8	7.8
V3	20	-	31.200	-	21.840	-	3.120	-	6.240	666	71.9	1.8	0.8	3.6
**Comp. Effort**														
V1	20	160	112.500	112	78.750	16	11.250	32	22.500	1.203	185.40	2.5	0.8	7.9
**Generalizability**														
Left-outs	2	16	9.795	-	-	-	-	16	9.795	105	16.14	2.5	0.7	3.7
Unknown Device	1	8	4.898	-	-	-	-	8	4.898	52	8.07	2.6	1.3	3.5
Interpolation (V3)	20	-	31.200	-	24.960	-	2.059	-	4.181	666	71.9	1.8	0.8	3.6
Extrapolation (V3)	20	-	31.200	-	24.960	-	2.059	-	4.181	666	71.9	1.8	0.8	3.6

**Table 2:** Accuracy; datasets V1–V3; velocity errors in  m/s, distance errors in m.

Dataset	PDR $K_{g}$						ML-GP						RoNIN						C/RNN						Hybrid					
of 20 Subjects	MAE	MSE	RMSE	${\mathrm{CEP}}_{95}$	DEPM	DET	MAE	MSE	RMSE	${\mathrm{CEP}}_{95}$	DEPM	DET	MAE	MSE	RMSE	${\mathrm{CEP}}_{95}$	DEPM	DET	MAE	MSE	RMSE	${\mathrm{CEP}}_{95}$	DEPM	DET	MAE	MSE	RMSE	${\mathrm{CEP}}_{95}$	DEPM	DET
V1 valid.	2.01	6.31	2.51	4.88	0.348	977.00	0.82	1.17	1.08	2.16	0.077	21.05	0.84	1.94	1.39	2.76	0.011	37.21	0.44	0.52	0.72	1.44	0.018	18.71	0.23	0.14	0.37	0.72	0.022	9.46
V1 test	2.16	7.37	2.71	5.25	0.374	2103.89	0.89	1.38	1.17	2.37	0.088	136.95	0.88	2.15	1.46	2.86	0.013	460.47	0.47	0.59	0.77	1.49	0.019	130.59	0.25	0.16	0.40	0.77	0.022	15.65
V1 test w. LKF	1.06	1.55	1.25	2.15	0.435	1451.61	0.51	0.44	0.66	1.41	0.065	363.80	0.64	0.93	0.97	1.91	0.178	101.70	0.38	0.39	0.62	1.22	0.055	38.53	0.22	0.13	0.36	0.70	**0.001**	5.32
V2 valid.	1.84	5.38	2.32	4.51	0.337	817.31	0.71	0.90	0.95	1.91	0.064	14.89	0.61	1.04	1.02	1.98	0.004	10.90	0.40	0.43	0.66	1.27	0.022	10.69	0.20	0.11	0.33	0.63	0.024	3.19
V2 test	1.93	5.82	2.41	4.69	0.328	1806.20	0.77	1.04	1.02	2.05	0.074	76.96	0.64	1.15	1.07	2.07	0.005	206.69	0.42	0.48	0.69	1.33	0.022	66.70	0.22	0.12	0.35	0.67	0.023	8.38
V2 test w. LKF	0.99	1.35	1.16	2.01	0.410	1010.47	0.47	0.37	0.61	1.30	0.054	267.01	0.51	0.64	0.80	1.56	0.127	62.35	0.34	0.33	0.57	1.11	0.043	36.98	0.19	0.10	0.32	0.61	0.004	4.93
V3 valid.	-	-	-	-	-	-	0.63	0.70	0.83	1.76	0.036	26.77	0.50	0.71	0.84	1.65	0.030	31.81	0.25	0.17	0.41	0.77	0.005	14.95	**0.15**	**0.06**	**0.24**	**0.44**	0.013	8.62
V3 test	-	-	-	-	-	-	0.67	0.78	0.88	1.87	0.047	39.84	0.55	0.85	0.92	1.80	0.042	145.08	0.28	0.21	0.46	0.89	0.010	21.50	0.17	0.07	0.27	0.53	0.016	12.66
V3 test w. LKF	-	-	-	-	-	-	0.43	0.30	0.55	1.16	0.076	113.82	0.47	0.52	0.72	1.45	0.135	22.52	0.24	0.17	0.41	0.80	0.026	14.80	0.16	0.07	0.26	0.50	0.003	**3.75**

**Table 3:** Computational effort.

Method	$t_{\mathrm{train}}$ [h]		$t_{\mathrm{live}}$ [s]		$t_{\mathrm{live}}$ [Hz]	
	CPU	GPU	CPU	GPU	CPU	GPU
PDR	0.2	-	0.00073	-	1370	-
ML-GP	23.6	-	0.00203	-	493	-
RoNIN	11.2	8.1	0.01041	0.0147	96	68
C/RNN	6.9	4.9	0.00610	0.0069	164	145
Hybrid	15.6	10.5	0.01785	0.0277	56	36

**Table 4:** Accuracy; generalizability; velocity errors in  m/s; distance errors in m.

Dataset	PDR $K_{g}$						ML-GP						RoNIN						C/RNN						Hybrid					
	MAE	MSE	RMSE	${\mathrm{CEP}}_{95}$	DEPM	DET	MAE	MSE	RMSE	${\mathrm{CEP}}_{95}$	DEPM	DET	MAE	MSE	RMSE	${\mathrm{CEP}}_{95}$	DEPM	DET	MAE	MSE	RMSE	${\mathrm{CEP}}_{95}$	DEPM	DET	MAE	MSE	RMSE	${\mathrm{CEP}}_{95}$	DEPM	DET
Left-out A	2.10	6.94	2.63	5.09	0.376	460.19	0.76	1.03	1.01	2.09	0.082	99.75	0.57	0.90	0.95	1.82	0.035	3.97	0.29	0.23	0.48	0.91	0.027	2.00	0.18	0.08	0.29	0.55	0.026	1.26
Left-out B	2.06	6.64	2.58	5.03	0.362	442.94	0.78	1.07	1.03	2.10	0.071	86.58	0.57	0.90	0.95	1.85	0.061	6.87	0.30	0.23	0.48	0.91	0.022	3.38	0.18	0.08	0.29	0.54	0.023	2.07
Left-outs A+B	2.10	6.95	2.64	5.08	0.374	914.55	0.78	1.08	1.04	2.14	0.077	188.13	0.58	0.92	0.96	1.85	0.029	4.43	0.30	0.23	0.48	0.93	0.024	2.33	0.19	0.09	0.29	0.56	0.024	1.54
Unknown devices	2.32	7.21	2.89	5.43	0.516	506.44	0.83	1.21	1.16	2.38	0.077	101.42	0.73	0.98	1.06	2.12	0.093	12.44	0.32	0.35	0.52	1.02	0.032	5.21	0.21	0.12	0.32	0.67	0.028	3.44
Interpolation valid.	-	-	-	-	-	-	0.54	0.49	0.70	1.47	0.105	61.31	0.49	0.61	0.78	1.49	0.060	34.84	0.24	0.15	0.38	0.70	0.005	16.86	0.15	0.05	0.23	0.42	0.007	10.12
Interpolation test	-	-	-	-	-	-	0.53	0.47	0.68	1.46	0.089	104.44	0.52	0.66	0.82	1.58	0.064	75.19	0.26	0.16	0.40	0.76	0.007	36.41	0.16	0.06	0.24	0.44	0.006	21.872

**Table 5:** Accuracy; left-outs; velocity errors in  m/s, distance errors in m.

Dataset of Left-Outs	PDR $K_{g}$						ML-GP						RoNIN						C/RNN						Hybrid					
	MAE	MSE	RMSE	${\mathrm{CEP}}_{95}$	DEPM	DET	MAE	MSE	RMSE	${\mathrm{CEP}}_{95}$	DEPM	DET	MAE	MSE	RMSE	${\mathrm{CEP}}_{95}$	DEPM	DET	MAE	MSE	RMSE	${\mathrm{CEP}}_{95}$	DEPM	DET	MAE	MSE	RMSE	${\mathrm{CEP}}_{95}$	DEPM	DET
Walking	1.15	1.82	1.35	2.17	0.054	81.51	0.70	0.68	0.83	1.36	0.093	138.81	0.67	0.62	0.78	1.33	0.004	5.67	0.32	0.15	0.38	0.66	0.015	2.56	0.17	0.04	0.20	0.32	0.003	2.26
Jogging	1.27	2.18	1.48	2.38	0.049	141.02	0.76	0.80	0.90	1.48	0.048	138.59	0.73	0.73	0.86	1.46	0.003	9.73	0.35	0.17	0.42	0.72	0.009	2.60	0.19	0.05	0.21	0.35	0.000	1.08
Running	1.35	2.50	1.58	2.54	0.023	87.74	0.83	0.95	0.97	1.62	0.037	140.97	0.80	0.87	0.94	1.56	0.001	4.49	0.39	0.21	0.46	0.77	0.006	3.52	0.21	0.05	0.23	0.38	0.002	2.33
Random	1.38	2.56	1.60	2.56	0.037	105.69	0.97	0.93	0.96	1.60	0.054	153.55	0.83	0.92	0.93	1.58	0.026	75.11	0.41	0.23	0.48	0.79	0.009	21.46	0.21	0.06	0.25	0.43	0.004	11.47

## Appendix A. Parameterization of Velocity Estimators

Note that we highlight the parameters that enable the highest accuracy of each velocity estimator with bold numbers.

### Appendix A.1. Feature Set of Baseline II, ML with Gaussian Processes ML-GP

The set of of 6 *time domain features* is inspired by Bishop et al. [79] and consists of absolute energy, i.e., the absolute energy of the time series which is the sum over the squared values, maximum, minimum, mean, median, and variance. The set of 7 *frequency domain features* is inspired by Vathsangam et al. [81] and comprises spectral bandwidth, spectral flatness, i.e., the ratio of the geometric mean to the arithmetic mean of the magnitude spectrum of the signal, spectral roll-out, i.e., frequency below which a certain percentage of the total spectral energy lies, spectral center of gravity, i.e., the spectral centroid (mean), variance, skew, and kurtosis of the absolute Fourier transform spectrum. While all time domain features are straightforward to calculate, the frequency domain features require to apply a short-term Fourier transform (STFT) to each window and to extract the features from the STFT-like representation of the time-frequency distribution. The grid search also examined a *dimensionality reduction* (principal component analysis, PCA [86]) of all 13 features to reduce them to feature bundles that contribute most to the accuracy of the estimation.

### Appendix A.2. Grid search results of Baseline II, ML with Gaussian Processes ML-GP

ML-GP grid search parameters: Logistic Reg.: penalty ∈[l1,**l2**,elasticnet], tolerance ∈[0.0001,**0.001**,1.0], *C*∈[0.1,**1.0**,10], solver ∈[**saga**,newton-cg]; SVR Linear: *C*∈[1,10,**100**,1000]; SVR Poly.: *C*∈[1,10,**100**,1000], degree = **3** ∈[2:1:6]; SVR RBF: γ ∈[**0.001**,0.0001], *C*∈[1,10,**100**,1000]; CART: max. depth = **97** ∈[1:1:150], max. features = **27** ∈[1:1:30], max. leaf nodes = **15** ∈[1:1:20]; GP: kernel ∈[RBF, **Matern52**, rotational quadratic], RQ α∈[0.001,0.01,0.1,1.0,**2.0**:10], RQ length scale ∈[0.1,**1.0**,:,10], RBF length scale ∈[0.1,1.0,**3.0**,:,10], M52 length scale ∈[0.1,**1.0**,:,10], M52 ν∈[0.1,1.0,1.5,**2.0**,2.5,:,10]; For every 84 feature combinations and combinations of $f_{s}$ (50,**100**,200,400) and $N_{w}$ (64,**128**,256,512). The parameters are in line with findings from Rasmussen et al. [82].

### Appendix A.3. Grid search results of Baseline III, DL I, RoNIN

RoNIN grid search parameters: solver ∈[SGD, **Adam**, rmsprop]; $\beta_{1},\beta_{2}$ = **0.01**; momentum = **0.9**; initial learning rate (LR) = **0.05**∈[1.0:0.1:0.00001]; LR drop period ∈[0,**10**,50,100] epochs; LR drop rate = **0.9**; patience = **100** epochs; batch size ∈[128,256,512,**1024**,2048]; ResNet $F_{s}$ = d×N with *d*∈[1,**2**] dimensions and *N* = **7**∈[3,4,ߪ,9,10]; ResNet s = **1**×N with *N*∈[1,**2**,...,9,10]; shuffle ∈[no,**per epoch**]; gradient clipping = max(input); dp∈[10,20,**50**,75]%; For every combination of $f_{s}$ (50,**100**,200,400) and $N_{w}$ (64,128,**256**,512). The parameters are in line with findings from Startsev et al. [87].

### Appendix A.4. Grid search results of Baseline IV, DL II, C/RNN

C/RNN grid search parameters: solver: **Adam**; $\beta_{1},\beta_{2}$ = **0.01**; momentum = **0.9**; B/LSTM layers = **2**; LSTM cells per layer = **128**; initial learning rate, LR = **0.01**; LR drop period = **10** epochs; LR drop rate per epoch = **0.9**; patience = **100** epochs; batch size = **512**; shuffle ∈[no,**per epoch**]; gradient clipping = max(input); dp∈[10,20,**50**,75]%; CNN $F_{s}$ = d×N with *d* = **2** dimensions and *N* = **3**; CNN s = **1**×N with *N* = **2**; For every combination of $f_{s}$ (50,**100**,200,400) and $N_{w}$ (64,128,**256**,512).

### Appendix A.5. Grid search results of our novel approach, DL III, Hybrid

We evaluated different sample rates (50, **100**, 200, and 400 Hz) and window lengths (64, **128**, 256, and 512 Hz) on either 1D or **2D** SMVs (best configuration in bold). The input sequence length of $N_{w}$ = 128 holds a maximum of 1.28 s (128/100 Hz) of motion data. Hybrid grid search parameters: solver ∈[SGD, **Adam**, rmsprop]; $\beta_{1},\beta_{2}$ = **0.01**, momentum = **0.9**; B/LSTM layers = **2** [1,2,3,4], cells/layer = **128** [16,32,64,128,256], initial learning rate (LR) = **0.01**∈[1.0:0.1:0.00001]; LR drop period ∈[0,**10**,50,100] epochs; LR drop rate = **0.9**; patience = **100** epochs; batch size ∈[128,256,512,**1024**,2048]; ResNet $F_{s}$ = d×N with *d*∈[1,**2**] dimensions and *N* = **7**∈[3,4,…,9,10]; ResNet s = **1**×N with *N*∈[1,**2**,…,9,10]; shuffle ∈[no,**per epoch**]; gradient clipping = max(input); dp∈[10,20,**50**,75]%; For every combination of $f_{s}$ (50,**100**,200,400) and $N_{w}$ (64,128,**256**,512).
